# Vasculogenic tissue nanotransfection accelerates functional recovery after peripheral nerve injury

**DOI:** 10.1126/sciadv.aeb7631

**Published:** 2026-04-08

**Authors:** Ana I. Salazar-Puerta, Sara Kheirkhah, Jonathan P. Stranan, Hallie Harris, Nada Khattab, Megumi Fukuda, Grant Barringer, Carlos A. Vasquez-Martinez, Samuel Cortes, Neil Ott, William R. Lawrence, Devleena Das, Jordan T. Moore, Tiam M. Saffari, Juan D. Salazar-Gil, Diego Alzate-Correa, Junyan Yu, Kavya Dathathreya, Natalia Higuita-Castro, Tatiana Z. Cuellar-Gaviria, W. David Arnold, Amy M. Moore, Daniel Gallego Perez

**Affiliations:** ^1^ Department of Biomedical Engineering, The Ohio State University, Columbus, OH, USA.; ^2^ Department of Plastic and Reconstructive Surgery, The Ohio State University, Columbus, OH, USA.; ^3^ Biomedical Science, The Ohio State University, Columbus, OH, USA.; ^4^ Biophysics Program, The Ohio State University, Columbus, OH, USA.; ^5^ Department of Neurosurgery, The Ohio State University, Columbus, OH, USA.; ^6^ Department of Physical Medicine and Rehabilitation, University of Missouri, Columbia, MO, USA.; ^7^ Department of Surgery, The Ohio State University, Columbus, OH, USA.

## Abstract

Peripheral nerve injuries (PNIs) remain a major clinical challenge, often leaving patients with lifelong sensory, motor, and functional impairments despite surgical repair. While gene and cell therapies hold promise, their translation has been hampered by the lack of safe, efficient, and targeted delivery strategies. Here, we introduce vasculogenic tissue nanotransfection (TNT) as a nonviral, reprogramming-based therapeutic platform to enhance nerve regeneration to augment surgical reconstruction. This one-time, millisecond-scale intervention reprograms resident cells in situ toward a vasculogenic phenotype, fostering neovascularization and vascular remodeling to support axonal regeneration. Through integrated in vitro screening and in vivo validation, we identified an optimized formulation of vasculogenic genes (*Etv2*, *Fli1*, and *Foxc2*; *EFF*) that maximized reprogramming efficiency and regenerative potential. In a long-segment nerve defect model reconstructed with isografts, TNT-mediated delivery of *EFF* markedly improved functional recovery, including grip strength and muscle contractility, accompanied by increased vascular density and myelinated axon counts. Together, these findings establish TNT-mediated vasculogenic reprogramming as a transformative adjunct to surgical repair of PNIs, offering a clinically translatable strategy to accelerate nerve regeneration and restore function.

## Teaser

A nonviral gene-reprogramming strategy enhances vascular support to promote functional recovery after nerve injury.

## INTRODUCTION

Peripheral nerve injuries (PNI) account for 2.8% of traumatic injuries in the United States, with an annual incidence of 13 to 23 cases per 100,000 people (*1*, *2*). These injuries impose a substantial financial burden, with $150 billion spent annually in the United States (*3*). While the peripheral nervous system has the inherent ability to regenerate, limitations of axonal regeneration exist including a slow and inefficient regeneration rate of 1 to 2 mm/day, the inability and inefficiency of axons to regenerate across a nerve gap without surgical intervention, and the time sensitivity of denervated motor and sensory end organs (*4*) . Thus, PNI can lead to substantial morbidity and disability, resulting in long-term consequences such as impaired motor and sensory function and/or chronic pain (*5*).

After a nerve injury, the goal of nerve reconstruction is to reestablish the nerve continuity with a tension-free nerve repair. However, if there is tension on the coaptation or if there is a segmental nerve gap, then surgical nerve reconstruction with nerve grafting can be performed. In this setting, most commonly, a patient’s own redundant and dispensable sensory nerve is used to bridge the segmental defects (*6*). Autografts provide a scaffold that supports nerve repair and regeneration while preserving essential cellular, molecular, and structural components, including Schwann cells, endothelial cells, extracellular matrix, and vasculature (*4*). Since they originate from the patient, autografts are not subjected to immune rejection; however, limited donor supply, secondary surgery, and size mismatches remain clinical challenges (*7*). While this approach has shown promise, motor and sensory functional recovery remains less than ideal, with only 40 to 50% function regained (*8*). Despite advances in understanding nerve injury and regeneration, as well as improvements in microsurgical techniques, full functional recovery remains elusive for most patients affected by PNI.

Novel strategies integrating cellular and molecular approaches are needed to advance PNI management by accelerating axon regeneration and reinnervation or delaying degeneration until surgical reconstruction can be performed, ultimately supporting timely synapse formation and functional recovery. Studies in the central nervous system (CNS) highlight the strong synergy between neurogenesis and angiogenesis (*9*, *10*). Similarly, PNI repair relies on this natural synergy, as newly formed blood vessels serve as scaffolds that support axonal growth and tissue regeneration (*11*). Current strategies focused on delivering neurotrophic factors such as nerve growth factor (NGF) and glial cell line–derived neurotrophic factor or angiotrophic factors such as vascular endothelial growth factor (VEGF), and fibroblast growth factor 2, alone or in combination, have faced limitations due to their short half-life and rapid clearances (*12*, *13*). Gene therapies have shown to promote revascularization and axonal growth but often require viral vectors, posing risks such as immune system activation, oncogenic potential, and cargo size constraints, limiting their clinical implementation (*14*). Likewise, while cell therapies show promise for regeneration, most of these rely on progenitor-like/stem-like cells (e.g., endothelial progenitor cells), which are scarce, difficult to isolate, and associated with risks such as uncontrolled differentiation, tumorigenesis, genetic anomalies, and immune reactions (*15*).

Recent advances in cell transdifferentiation have the potential to enable patient-specific therapies by converting abundant and readily available cells (e.g., fibroblasts) into other functional cell types, avoiding the need for induced pluripotency and its associated risks (*16*, *17*). Various methods have been explored to deliver specific transcription factors (TFs) to drive this direct conversion (*18*). We have previously demonstrated that a combination of TFs including Ets variant 2 (*Etv2*), Friend leukemia virus integration 1 (*Fli1*), and Forkhead box C2 (*Foxc2*), collectively referred to as *EFF*, can induce vasculogenic conversion in somatic cells and sustain vascular integrity over time. *Etv2* acts as a pioneer TF that initiates endothelial gene programs (*19*), *Fli1* supports endothelial maturation and maintenance (*20*), and *Foxc2* contributes to vascular remodeling through downstream VEGF-Notch signaling (*21*). Our group has developed tissue nanotransfection (TNT), a nonviral technology for driving multi-pronged gene and cell therapies directly within solid tissues (e.g., skin and peripheral nerves) (*22*, *23*). TNT operates by delivering molecular cargo through arrays of nanochannels integrated to microneedles. Upon application of a brief, focused electric field, the nanochannels induce transient pores in the cell membrane and electrophoretically drive charged cargo (e.g., plasmid DNA, microRNAs, and oligonucleotides) into underlying cells. The delivery depth, spatial distribution, and relative efficiency of this nanochannel-confined process have been quantitatively characterized in our prior work (*22*, *23*). This single-step approach enables in vivo provasculogenic conversions, thereby enhancing vascularization and promoting tissue repair and regeneration across different models.

Here, we report the use of vasculogenic TNT as a promising strategy to enhance regenerative outcomes following PNI and surgical reconstruction with nerve isografts. By inducing localized neovascularization, vasculogenic TNT fosters a proregenerative microenvironment that supports axonal growth. We demonstrate that TNT enables vasculogenic reprogramming of nerve-resident cells, establishing a vascular scaffold that can guide axonal regeneration and enhance functional recovery. Through optimization of *EFF* ratios, we were able to significantly improve vasculogenic conversions both in vitro and in vivo, which led to improved outcomes when deployed in combination with surgical reconstruction of a long segmental defect. Lineage tracing studies suggest that vasculogenic conversions are driven by nerve fibroblasts, underscoring the potential of TNT-mediated vasculogenic signaling to drive nerve repair. Collectively, these findings establish vasculogenic TNT as a powerful, nonviral platform for coordinating vascular and neural regeneration, offering a previously unknown therapeutic avenue for the repair of PNI.

## RESULTS

### Modulating the *EFF* ratio alters vasculogenic reprogramming outcomes in fibroblast cultures

**Fig. 1.**
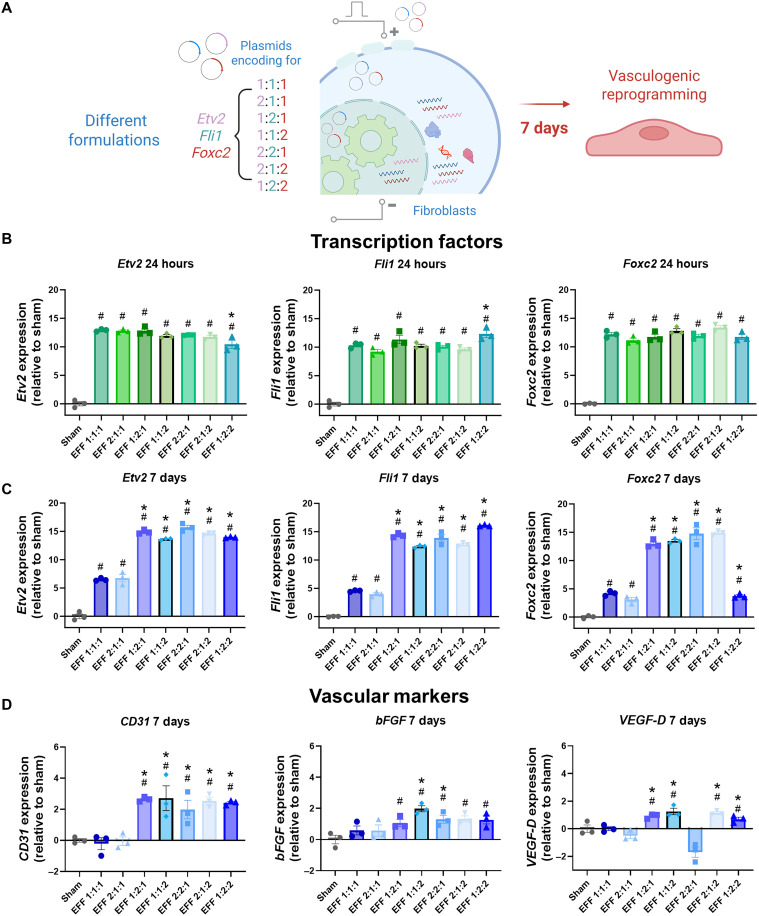
In vitro overexpression of different formulations of *EFF* initiates a vasculogenic reprogramming cascade in fibroblasts. (**A**) Schematic diagram illustrating the vasculogenic reprogramming initiated by the electrotransfection of fibroblasts with different mass ratios of expression plasmids for *Etv2*, *Fli1*, and *Foxc2* (*EFF*), leading to transcriptional and phenotypic changes associated with the vasculogenic lineage after 7 days. (**B**) qRT-PCR analysis showing increased gene expression of *Etv2*, *Fli1*, and *Foxc2* in all *EFF* formulations 24 hours after transfection. (**C**) Gene expression analysis 7 days posttransfection indicates persistent overexpression of *EFF* in all formulations compared to the sham-transfected cells; however, some *EFF* formulations (1:2:1, 1:1:2, 2:2:1, and 2:1:2) showed higher expression compared to *EFF* 1:1:1. (**D**) Expression of vascular markers (*CD31*, *VEGF-D*, and *bFGF*) was found as early as 7 days posttransfection for some *EFF* formulations (1:2:1, 1:1:2, 2:2:1, 2:1:2, and 1:2:2) compared to the sham and *EFF* 1:1:1 groups (*n* = 3). All error bars are shown as SEM. # denotes significant difference respect to the sham with a *P* value < 0.05. * denotes significant difference with respect to *EFF* 1:1:1 with a *P* value < 0.05, one-way ANOVA. Created in BioRender. A. Salazar (2026); https://BioRender.com/yn3s2jn.

Fibroblast cultures were electrotransfected with expression plasmids encoding for vasculogenic TFs *Etv2*, *Fli1*, *Foxc2* (*EFF*)*.* Electrotransfected fibroblasts cultures with a sham/empty pCMV6 vector were used as control (i.e., sham). Previous studies from our group and others have shown that delivery of *EFF* at a 1:1:1 ratio leads to de novo formation of vascular tissue (*22*–*25*). Given that reprogramming TFs are known to function in a context-dependent manner (*26*, *27*), it is reasonable to anticipate that varying the stoichiometries of *EFF* could yield distinct outcomes. Therefore, to identify the optimal ratio for enhancing vasculogenic reprogramming, different *EFF* stoichiometries (1:1:1, 2:1:1, 1:2:1, 1:1:2, 2:2:1, 2:1:2, and 1:2:2) were tested in vitro (Fig. 1A). Gene expression was initially evaluated across all conditions, confirming that *EFF* were robustly overexpressed 24 hours posttransfection compared to sham-transfected cells (Fig. 1B). Moreover, the analysis at 7 days posttransfection revealed that *EFF* expression remained elevated across most conditions (average expression level ~15), whereas it declined sharply in the conventional 1:1:1 *EFF* group (Fig. 1C). These findings suggest that while increasing the mass of individual plasmids within the *EFF* mixture does not necessarily boost early expression at 24 hours, it plays a critical role in sustaining the expression of all factors over time, thus preventing the marked decline observed with the 1:1:1 formulation. To evaluate vasculogenic reprogramming, the expression of vascular markers such as cluster of differentiation 31 (CD31), basic fibroblast growth factor (bFGF), and vascular endothelial growth factor-D (VEGF-D) was assessed 7 days post-*EFF* delivery. The results showed that only certain formulations exhibited significantly higher expression of these markers compared to the sham and 1:1:1 *EFF* groups. Among these, the *EFF* 1:2:1, 1:1:2, 2:2:1, 2:1:2, and 1:2:2 showed enhanced signs of vasculogenic conversion (Fig. 1D).

### Mass ratio–dependent delivery of *EFF* via TNT modulates vasculogenic reprogramming in a sciatic nerve injury model

**Fig. 2.**
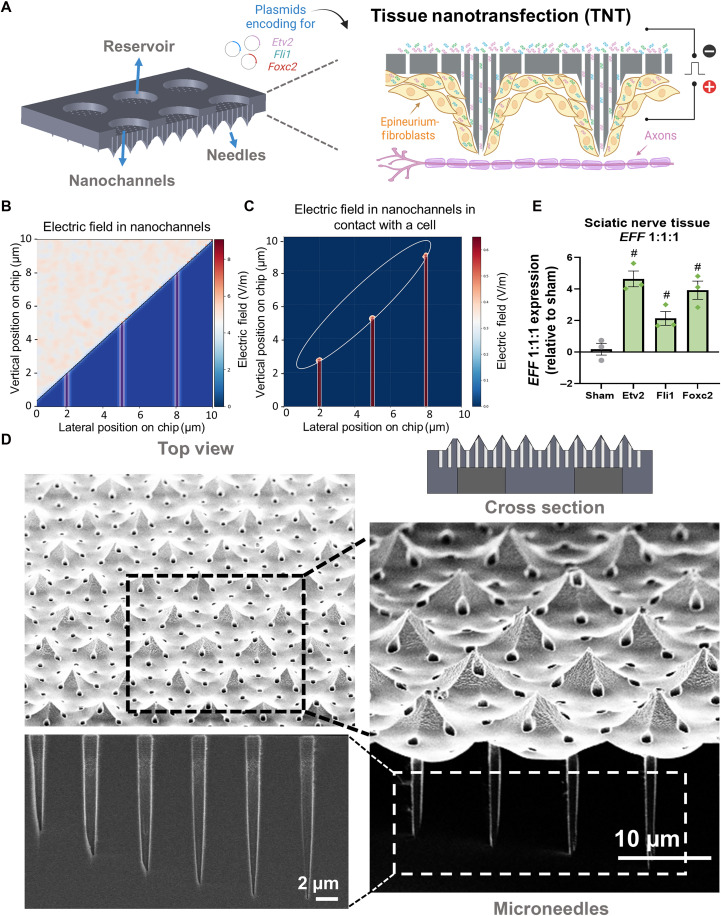
TNT mediates reprogramming factor delivery. (**A**) Schematic representation of the TNT chip, illustrating the location of the plasmid reservoir, nanochannels, and microneedles and depicting their interaction with the cells in direct contact with the device surface. Finite element method (FEM) modeling illustrates the electric field distribution in the nanochannels, highlighting DNA migration pathways in regions of high field intensity. (**B**) Electric field distribution across nanochannels showing intensity gradients (red to blue) at the sciatic nerve tissue interface. Higher intensities appear as vertical bands, demonstrating controlled field penetration necessary for targeted cell membrane interaction. (**C**) Electric field penetration pathway (white outline) with red vertical marks indicating threshold points where nanoporation occurs. This visualization confirms how TNT technology achieves precise gene transfection by creating optimal electroporation zones while preserving surrounding nerve tissue. (**D**) SEM micrographs showing the top view of the surface, cross section of the chip, and arrays of nanochannels extending beyond the planar Si surface (needle pattern) that interact with cells in the tissue. (**E**) Gene expression analysis of sciatic nerve tissue reveals overexpression of *EFF* (1:1:1) compared to sham-treated tissue 24 hours after TNT intervention, confirming the feasibility of using TNT-meditated gene delivery to nerve tissue. (*n* = 3). All error bars are shown as SEM. # denotes significant difference respect to the sham with a *P* value < 0.05, one-way ANOVA. Created in BioRender. A. Salazar (2026); https://BioRender.com/yn3s2jn.

TNT enables efficient, safe, and direct nanochannel-based delivery of molecular cargo into solid tissues through a one-time intervention lasting only milliseconds. This is achieved through the application of a focused electric field through the nanochannels, which porates the cell membranes and drives charged molecular cargo such as DNA into the cells via electrophoresis. The nanochannel-confined electric fields restrict poration to the localized tissue area in contact with the device, thereby preventing the high-intensity field exposure that can damage nerve tissue (Fig. 2, A to C). TNT devices were fabricated as previously described (*22*). Briefly, a combination of photolithography and dry etching was used to manufacture Si chips with microscale needles on the surface and through-thickness nanochannels, enabling the transfer of plasmids from the microreservoirs into the cells via a combination of nanoscale poration and electrophoresis. Characterization was conducted by scanning electron microscopy (SEM) to assess the structural and geometrical integrity of the devices (Fig. 2D). To validate TNT-mediated gene delivery, we first transfected healthy sciatic nerve tissue, demonstrating successful incorporation of the TF plasmids. Gene expression analysis at 24 hours post-TNT confirmed successful delivery of *EFF* compared to sham-transfected tissue (Fig. 2E and fig. S1A).

**Fig. 3.**
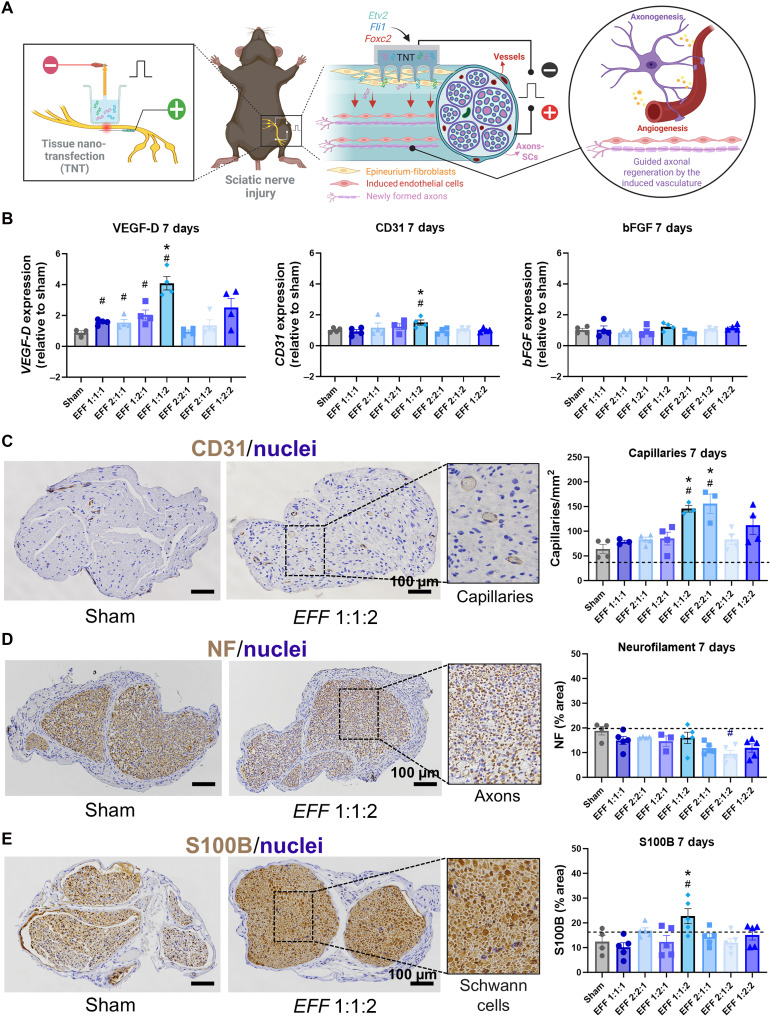
TNT with *EFF* improves vascular tissue formation in a mouse model of crush injury. (**A**) Schematic representation of the TNT intervention and the in vivo experiment using a crush injury mouse model followed by TNT with *EFF* to the sciatic nerve to induce the formation of vascular tissue and contribute to axonal growth and regeneration. The sciatic nerve is surgically exposed, crushed, and the surface of the TNT platform is placed in direct contact with the epineurium (outermost layer of the sciatic nerve). The genetic cargo is driven into the cells via a combination of nanoscale electroporation and electrophoretic motility after applying a pulsed electric field (200 V, 10-ms pulses, and 10 pulses) across electrodes. A positive electrode is placed underneath in direct contact with the nerve, and the negative electrode inside the reservoir interacting with the DNA solution. (**B**) Successful expression of the vascular marker *VEGF-D* was found 7 days post-TNT in certain conditions (*EFF* 1:1:1; 2:1:1, 1:2:1, and 1:1:2), and *CD31* was only up-regulated in the *EFF* 1:1:2 group compared to *EFF* 1:1:1 and sham-transfected tissue. Histological analysis on cross sections of the sciatic nerve tissue shows (**C**) a significant increase in capillary density by the expression of *CD31* in the *EFF* 1:1:2 and 2:2:1 formulations compared to *EFF* 1:1:1, sham-treated, and healthy tissue (dashed line) groups; (**D**) no significant differences were found in the expression of the axonal marker NF with levels comparable to healthy tissue; and (**E**) an elevated expression of S100B, a Schwann cell marker, was found only in the *EFF* 1:1:2 group compared to all other groups (*n* = 3 to 5). All error bars are shown as SEM. # denotes significant difference with respect to sham with a *P* value < 0.05, and * denotes significant difference with respect to *EFF* 1:1:1 with a *P* value < 0.05, one-way ANOVA. Created in BioRender. A. Salazar (2026); https://BioRender.com/yn3s2jn.

To assess whether changes in *EFF* composition impact vasculogenic conversions in vivo, we applied TNT-mediated delivery of *EFF* at different mass ratios to sciatic nerves following crush injury in 8- to 10-week-old C57BL/6 mice. After sciatic nerve injury, TNT was performed using the vasculogenic *EFF* cocktail, applied to the outermost layer of the nerve (epineurium), which is composed of dense irregular connective tissue (e.g., collagen and fibroblasts). Nerve tissue was evaluated 7 and 14 days postintervention (Fig. 3A). Similar to in vitro experiments, various *EFF* mass ratios were tested to assess reprogramming outcomes. TNT with sham/empty plasmids served as a control (sham). Gene expression analysis of vascular markers (e.g., CD31, bFGF, and VEGF-D) evaluated 7 days post-TNT revealed that only certain conditions significantly up-regulated VEGF-D compared to the sham group. These included *EFF* 1:1:1, 2:1:1, 1:2:1, and 1:1:2. However, only the *EFF* 1:1:2 group showed significant overexpression CD31 (Fig. 3B). Histological analyses of the nerve at day 7 postcrush and TNT, using chromogenic staining, showed that *EFF* 1:1:2 and 2:2:1 groups exhibited a significant increase in capillary density compared to the conventional *EFF* 1:1:1, sham-treated, and healthy tissue (dashed line) groups (Fig. 3C). Immunostaining for an axonal marker, neurofilament (NF) heavy light chain, showed no significant differences in expression across all formulations, with levels comparable to healthy tissue (Fig. 3D). Notably, the *EFF* 1:1:2 group demonstrated elevated expression of S100B, a Schwann cell marker, compared to all other groups (Fig. 3E), highlighting enhanced Schwann cell activation, a critical step for initiating the inflammatory and regenerative cascades necessary for nerve repair (*28*). While S100B is maintained at low levels under physiological conditions, its local up-regulation after injury triggers neurotrophic effects by recruiting macrophages, promoting cytokine release, and establishing a bioactive environment that supports axonal regrowth and remyelination (*28*, *29*). Consistent with these findings, histological analyses at day 14 postcrush and TNT, using immunofluorescence staining, showed that the *EFF* 1:1:2 formulation resulted in a significantly higher number of capillaries compared to all other *EFF* formulations, sham-treated, and healthy tissue (fig. S2A). In addition, the *EFF* 1:1:2 group exhibited greater expression of NF compared to other groups (fig. S2B).

### Whole transcriptome analysis reveals early activation of vasculogenic programs in vivo and in vitro

**Fig. 4.**
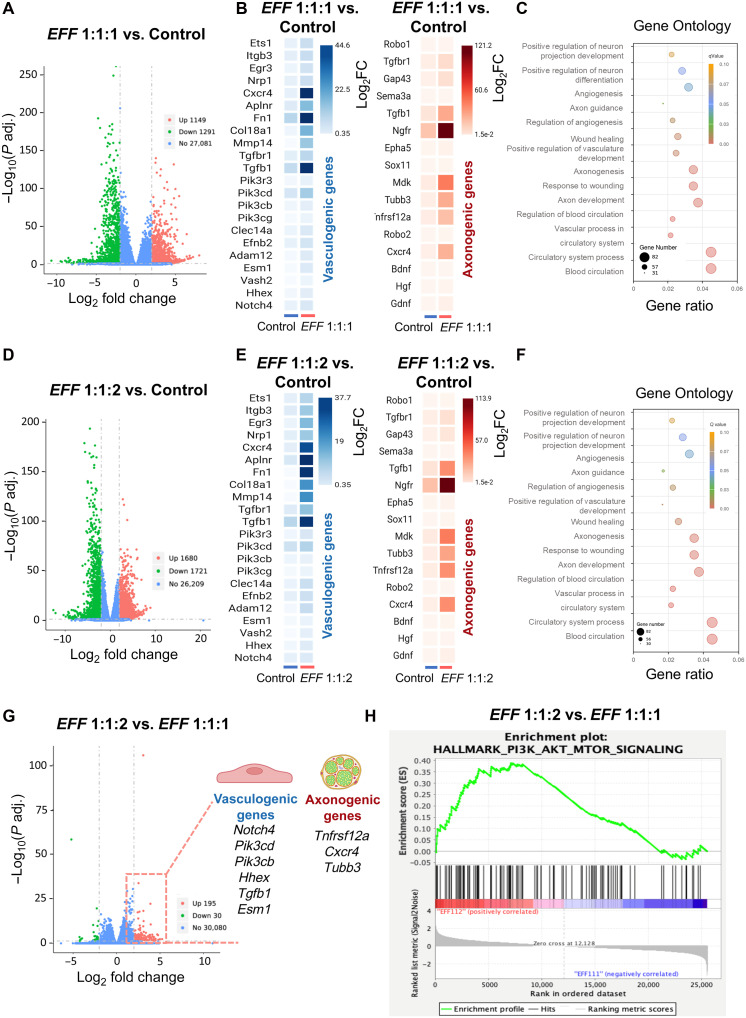
Transcriptional landscape reveals the impact of vasculogenic reprogramming 7 days post-crush injury and TNT treatment. RNA-seq analysis was performed on sciatic nerve tissue 7 days after crush injury and TNT comparing *EFF* 1:1:1 and *EFF* 1:1:2 TNT to healthy tissue (control). Volcano plots illustrating significantly up-regulated genes in red, down-regulated genes in green, and not differentially expressed in blue. (**A**) Up-regulated and down-regulated genes in the *EFF* 1:1:1 group compared to control. (**B**) Clustered heatmap shows the most differentially expressed vasculogenic and axonogenic genes between *EFF* 1:1:1 compared to control. (**C**) GO analysis show the BPs related to angiogenesis and axonogenesis enriched in the *EFF* 1:1:1 compared to control. (**D**) Volcano plot showing *EFF* 1:1:2 group compared to control, (**E**) the clustered heatmap for the *EFF* 1:1:2 TNT-treaded tissue compared to control, and (**F**) the GO analysis of *EFF* 1:1:2 groups tissue compared to control. (**G**) Direct comparison between the two *EFF* formulations revealed the up-regulation of vasculogenesis and axonogenesis-related genes in *EFF* 1:1:2 compared to the conventional *EFF* 1:1:1 TNT-treated tissue. (**H**) Last, GSEA plots show the significant activation of the PIK3 AKT/mTOR signaling in the *EFF* 1:1:2 group compared to *EFF* 1:1:1. Genes with Log_2_FC ≥ ∣2∣ and adjusted *P* value ≤ 0.05 were considered significantly differentially expressed.

On the basis of results from both in vitro and in vivo experiments, *EFF* 1:1:2 emerged as a leading formulation, ranking among the top candidates in in vitro studies and subsequently solidifying these findings in a mouse model of crush injury by driving increased vascularity and axonal density. Whole transcriptome analyses using RNA sequencing (RNA-seq) were performed to further investigate the transcriptional landscape of sciatic nerve tissue 7 days post-crush injury and TNT intervention using a standard Illumina next-generation sequencing platform pipeline. All analyses were conducted with thresholds of log_2_ fold change (Log2FC) ≥ ∣2∣ and an adjusted *P* value < 0.05. The differential expression analysis revealed substantial transcriptional changes among *EFF* 1:1:1, the selected 1:1:2 formulation, and sham-TNT group, relative to healthy tissue that was not subjected to crush injury or TNT (fig. S3A). Analyses for the *EFF* 1:1:1 formulation are shown in Fig. 2 (A to C), while analyses for the *EFF* 1:1:2 formulation are shown in Fig. 2 (D to F). A direct comparison between the *EFF* 1:1:1 and *EFF* 1:1:2 formulations is presented in Fig. 2 (G and H). We identified 1149 significantly up-regulated and 1291 down-regulated genes in the *EFF* 1:1:1 group compared to healthy tissue (Fig. 4A). Similarly, 1680 up-regulated and 1721 down-regulated genes were identified in the *EFF* 1:1:2 group (Fig. 4D). The expression profiles of vasculogenic and axonogenic genes of TNT-mediated delivery of *EFF* (1:1:1 and 1:1:2) compared to healthy tissue revealed distinct transcriptional shifts (Fig. 4, B and E). Similarly, heatmaps demonstrated clear differences in the expression patterns between healthy control and *EFF-*treated groups, particularly in genes associated with vasculogenesis and axonogenesis (fig. S3B). Direct comparison between the two *EFF* formulations revealed 195 genes up-regulated in the *EFF* 1:1:2 group, including vasculogenesis-associated genes (*Notch4*, *Pik3cd*, *Pick3cb*, *Hhex*, *Tgfb1*, and *Esm1*) and axonogenesis-related genes (*Tnfrsf12a*, *Cxcr4*, and *Tubb3*), suggesting a potential role for vasculogenic reprogramming in facilitating axonal repair and regeneration processes (Fig. 4G).

Gene Ontology (GO) enrichment analysis further confirmed strong enrichment of biological process (BP) related to angiogenesis, blood circulation, vascular remodeling, neuron differentiation, axon development, and axonogenesis in both *EFF* 1:1:1 and 1:1:2 groups (Fig. 4, C and F). Gene Set Enrichment Analysis (GSEA) was also evaluated to provide deeper insights, revealing significant activation of pathways linked to angiogenesis, cellular plasticity, neuroprotection, and endothelial and axon regeneration in the *EFF* 1:1:2 group compared to *EFF* 1:1:1 (Fig. 4H) and healthy tissue (fig. S3C). Notably, processes and signaling pathways such as angiogenesis; epithelial-mesenchymal transition (EMT); complement activation; interleukin-6 (IL-6) Janus kinase (Jak) signal transducers and activators of transcription 3 (Stat3); and phosphatidylinositol 3-kinase PIK3 AKT mechanistic target of rapamycin (mTOR) signaling were enriched when comparing *EFF* 1:1:2 to *EFF* 1:1:1, sham-TNT, and healthy tissue, reinforcing the functional relevance of these transcriptional changes. When comparing the sham-TNT group that underwent both crush injury and TNT without any therapeutic cargo with the healthy tissue, 1100 up-regulated genes were found compared to the healthy control, indicating that the crush and TNT interventions alone lead to transcriptional changes (fig. S3D). However, 117 genes were found up-regulated when comparing *EFF* 1:1:2 and sham-TNT (fig. S3E), emphasizing the impact of the *EFF* 1:1:2 cargo on gene expression. Although no differentially expressed genes (DEGs) met the threshold of Log2FC ≥ ∣2∣ when comparing *EFF* 1:1:1 with sham-TNT, GSEA revealed the enrichment of key BPs and signaling pathways, including transforming growth factor–β signaling, angiogenesis, EMT, and inflammatory response (fig. S3F). Together, these results support the premise that TNT-based delivery of *EFF* 1:1:2 drives vasculogenic and axonogenic processes in injured nerve tissue.

**Fig. 5.**
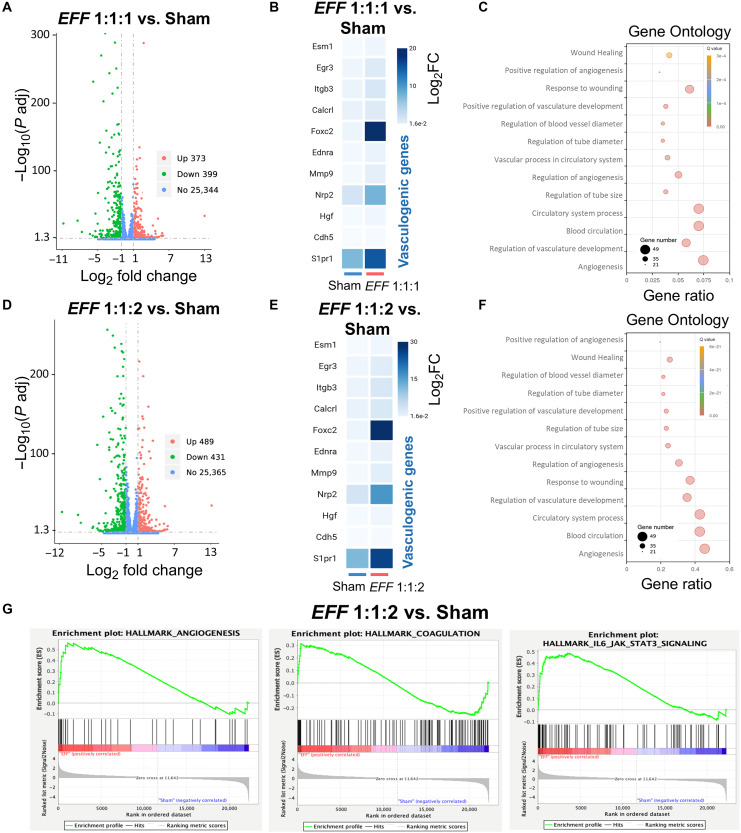
RNA-seq uncovers the transcriptional response to vasculogenic reprogramming of fibroblasts. RNA-seq analysis from fibroblasts 24 hours after transfection comparing the *EFF* 1:1:1 and *EFF* 1:1:2 formulations to sham-transfected cells. For each pair-wise comparison DEGs were identified and represented as follows. Volcano plots illustrating significantly up-regulated genes in red, down-regulated genes in green, and not DEGs in blue. (**A**) Transcriptomic profiles for *EFF* 1:1:1 compared to sham-transfected cells. (**B**) Most differentially expressed vasculogenic genes between *EFF* 1:1:1 compared to the sham group are shown in the clustered heatmap. (**C**) GO analysis revealed significant enrichment in BPs related to angiogenesis, vasculogenesis, and blood vessel formation in the *EFF* 1:1:1 compared to sham-transfected cells. Likewise, (**D**) volcano plot showing the DEGs for the *EFF* 1:1:2 group compared to sham-transfected cells. (**E**) Clustered heatmap showing vasculogenic genes expressed in the *EFF* 1:1:2–treated cells compared to the sham group. (**F**) Biological processes related to angiogenesis, vasculogenesis, and blood vessel formation in the *EFF* 1:1:2 compared to sham-transfected cells by GO analyses. (**G**) GSEA enrichment plot highlights angiogenesis, coagulation, and IL-6 Jak Stat3 signaling pathways enriched in the *EFF* 1:1:2 compared to sham group. Genes with Log_2_FC ≥ ∣2∣ and adjusted *P* value ≤ 0.05 were considered significantly differentially expressed.

After establishing that *EFF* 1:1:2 was superior to the conventional *EFF* 1:1:1 formulation in promoting vascular and neural regeneration in vivo, we next sought to further investigate early transcriptomic changes induced by this optimized cocktail in vitro. Because the in vivo RNA-seq captures transcripts from multiple cell types, we performed RNA-seq on isolated fibroblast cultures to specifically characterize fibroblast-driven transcriptional responses. To this end, RNA-seq was performed on fibroblasts 24 hours post-*EFF* delivery to gain insight into the immediate cellular responses and molecular pathways activated early during the initial stages of vasculogenic reprogramming. These experiments compared the *EFF* 1:1:1 and the selected *EFF* 1:1:2 formulations to sham-transfected cells and to a group of naïve fibroblasts (control) that were not subjected to electrotransfection (fig. S4A). The analyses for the *EFF* 1:1:1 group are presented in (A) to (C) and the analyses for the *EFF* 1:1:2 are shown in (D) to (F). Differential expression analysis revealed a total of 373 significantly up-regulated and 399 down-regulated genes between the *EFF* 1:1:1 group compared to sham-transfected cells, as visualized in the volcano plot (Fig. 5A). Similarly, 489 significantly up-regulated and 431 down-regulated genes were detected when comparing the *EFF* 1:1:2 group with the sham (Fig. 5D). The heatmaps show that the DEGs associated with vasculogenesis were highly expressed in the *EFF* groups compared to sham-transfected cells (Fig. 5, B and E, and fig. S4B). Moreover, a clear difference in the expression of the *Foxc2* gene was observed, with significantly higher levels in the *EFF* 1:1:2 compared to the 1:1:1 ratio, as expected due to the double mass ratio of this factor in the 1:1:2 cocktail. In addition, key genes such as *Notch2*, *Notch4*, *Dll1*, and *Dll4* and vasculogenic genes such as *Nrp2* and *S1pr1* were also found more up-regulated in the 1:1:2 compared to the 1:1:1 group. These genes play critical roles in regulating endothelial cell function and vascular patterning during angiogenesis (*Nrp2*) (*30*), as well as regulating endothelial cell barrier function, promoting vascular maturation, and driving sprouting angiogenesis (*S1pr1*) (*31*). Volcano plots showing the DEGs of *EFF* 1:1:1 (fig. S4C) and 1:1:2 (fig. S4F) with substantial differences in the number of up-regulated (~2800) and down-regulated (~2700) genes compared to control cells. Similarly, heatmap clustering of all DEGs demonstrated distinct clustering of samples between groups, suggesting robust transcriptional differences when comparing the *EFF* groups to control cells (fig. S4, D and G), and the genes associated with vasculogenesis in the *EFF* groups compared to the control group (fig. S4, E and H). This clustering across all comparisons reflects underlying shifts in the transcriptional landscape necessary to support the provasculogenic phenotypic changes driven by the *EFF* cocktails. In addition, the GO analysis of DEG revealed significant enrichment of BP related to angiogenesis, vasculogenesis, and blood vessel formation in the *EFF* 1:1:1 and 1:1:2 groups compared to sham-transfected cells (Fig. 5, C and F). Among the most significantly enriched BP, angiogenesis, regulation of vasculature development, and circulatory system were highly represented, highlighting the potential functional impact of these transcriptional changes. Notably, the number of genes associated with each BP varied between groups. For instance, the angiogenesis BP included 56 enriched genes in the *EFF* 1:1:2 group compared to 49 genes in the *EFF* 1:1:1. Similarly, the regulation of vasculature development BP comprised 41 genes in *EFF* 1:1:2 compared to 38 genes in the *EFF* 1:1:1, and in the circulatory system BP, 52 genes enriched in the *EFF* 1:1:2 versus 46 genes in *EFF* 1:1:1, suggesting differences in the extent of pathway activation between conditions. GSEA was performed to further investigate the processes and signaling pathways enhanced in the DEGs, which revealed significant enrichment of pathways related to angiogenesis, EMT, IL-6 Jak Stat3 signaling, coagulation, and apical junction in the *EFF* 1:1:2 group compared to the sham group (Fig. 5G), further confirming our observations regarding the initiation of a vasculogenic reprogramming process. Together, these findings further validate the transcriptional changes associated with vasculogenic conversion in fibroblasts. Notably, these commitments emerge as early as 24 hours post-*EFF* transfection, well before any detectable morphological or functional changes, underscoring the rapid and robust transcriptional response that precedes full cellular reprogramming.

### Lineage tracing reveals sciatic nerve fibroblasts as the primary target of TNT-driven vasculogenic conversions

**Fig. 6.**
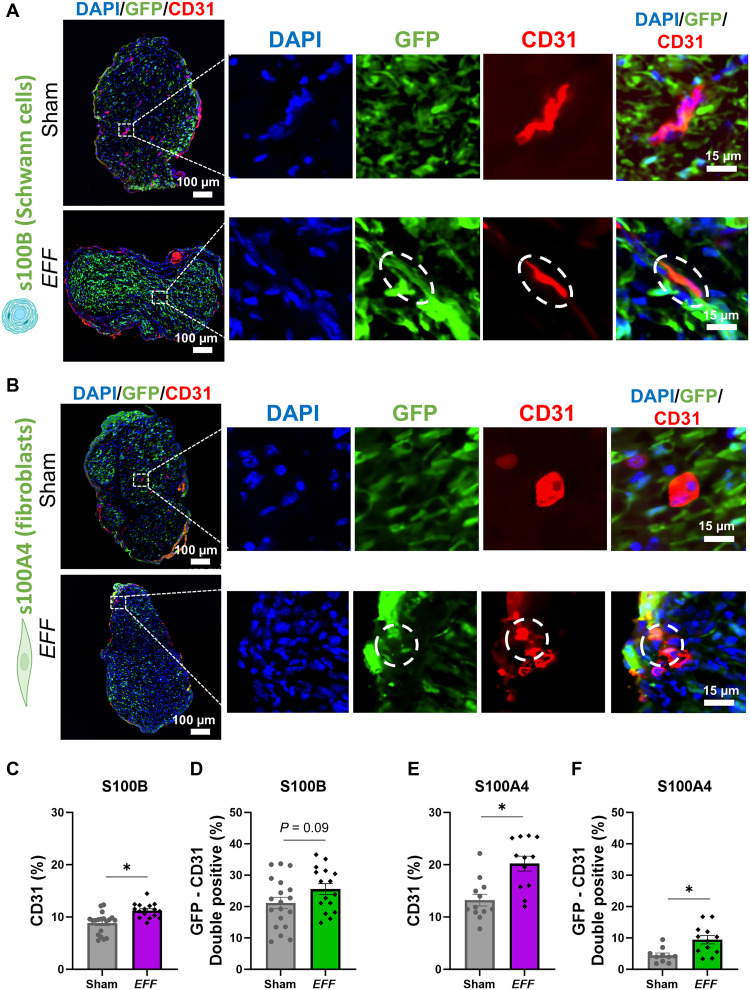
Fibroblasts are the primary source of induced endothelial cells in sciatic nerve tissue after reprogramming via vasculogenic TNT. Immunofluorescence micrographs represent the linage tracing analysis of sciatic nerve tissue 7 days after crush injury followed by *EFF* or sham TNT, showing GFP linage tracer expression in transgenic mouse models to identify cells originating from (**A**) Schwann cells (S100B) or (**B**) fibroblasts (S100A4). (**C**) Histological analysis revealed increased CD31 expression in the *EFF* TNT-treated group compared to the sham group in the S100B-EGFP mouse model, demonstrating successful vasculogenic reprogramming. (**D**) However, there was little to no increase in coassociation of the lineage tracer for S100B (GFP, green) with the endothelial marker (CD31, red) and cell nuclei (DAPI, blue) compared to the sham group. (**E**) In contrast, the S100A4-EGFP mouse model showed significantly higher expression of CD31 in the *EFF* treatment compared to the sham group and (**F**) a significant increase in coassociation between GFP-labeled fibroblasts with CD31 and DAPI in response to *EFF* TNT, suggesting that fibroblasts are the predominant contributors to the newly formed vascular cell population induced by reprogramming. All error bars are shown as SEM; * denotes significant difference with respect to sham with a *P* value < 0.05, two-tailed *t*-test.

To identify the cell populations within the nerve bundle that are more likely to undergo vasculogenic reprogramming, we conducted lineage-tracing studies using two transgenic mouse models: B6;D2-Tg(S100B-EGFP)1Wjt/l (*32*) and B6.Cg-Tg(S100A4-EGFP)M1Egn/YunkJ (*33*). These models were selected to trace distinct cell populations in the sciatic nerve, specifically Schwann cells (S100B-EGFP) and fibroblasts (S100A4-EGFP), respectively (fig. S5). In these experiments, a surgical crush injury was induced in the sciatic nerve of each mouse model, followed by TNT-based delivery of the selected *EFF* formulation (1:1:2) or sham plasmids. Sciatic nerve tissue was harvested and processed for histological analysis 7 days postintervention to evaluate the coassociation of the endothelial marker (CD31) with the lineage tracer reporter [green fluorescent protein (GFP)] and cell nuclei [4′,6-diamidino-2-phenylindole (DAPI)]. In the S100B-EGFP mouse model, our results revealed an increased expression of CD31 in the *EFF* group compared to the sham group (Fig. 6, A and C), suggesting successful vasculogenic induction, as demonstrated previously. However, there was no significant increase in the coassociation of the GFP reporter for Schwann cell lineage–derived cells with CD31 in the *EFF* group compared to sham (Fig. 6, A and D), suggesting that Schwann cells may not be a major contributor to vasculogenic conversions in response to TNT of *EFF*. In contrast, in the S100A4-EGFP mouse model, we observed a significant increase in CD31 expression in the *EFF* group compared to sham (Fig. 6, B and E), along with a significant increase in the coassociation of the GFP reporter for the fibroblast lineage with CD31 (Fig. 6, B and F), suggesting that sciatic nerve fibroblasts are a primary source of vasculogenic conversions in response to TNT of *EFF*. These findings indicate that fibroblasts, rather than Schwann cells, are a major contributor to the newly formed endothelial cell population induced by vasculogenic reprogramming. This observation is consistent with our previous published work (*23*), in which we demonstrated that in vitro cultures of sciatic nerve fibroblasts exhibit greater vasculogenic plasticity compared to Schwann cells, as evidenced by the presence of vascular marker–positive cells in fibroblasts, which were absent in Schwann cell cultures 7 days after transfection with the vasculogenic cocktail. Together, these results further support the preferential reprogramming potential of fibroblasts in both in vitro and in vivo settings.

### TNT enhances functional recovery in a mouse model of segmental nerve defect with graft reconstruction

**Fig. 7.**
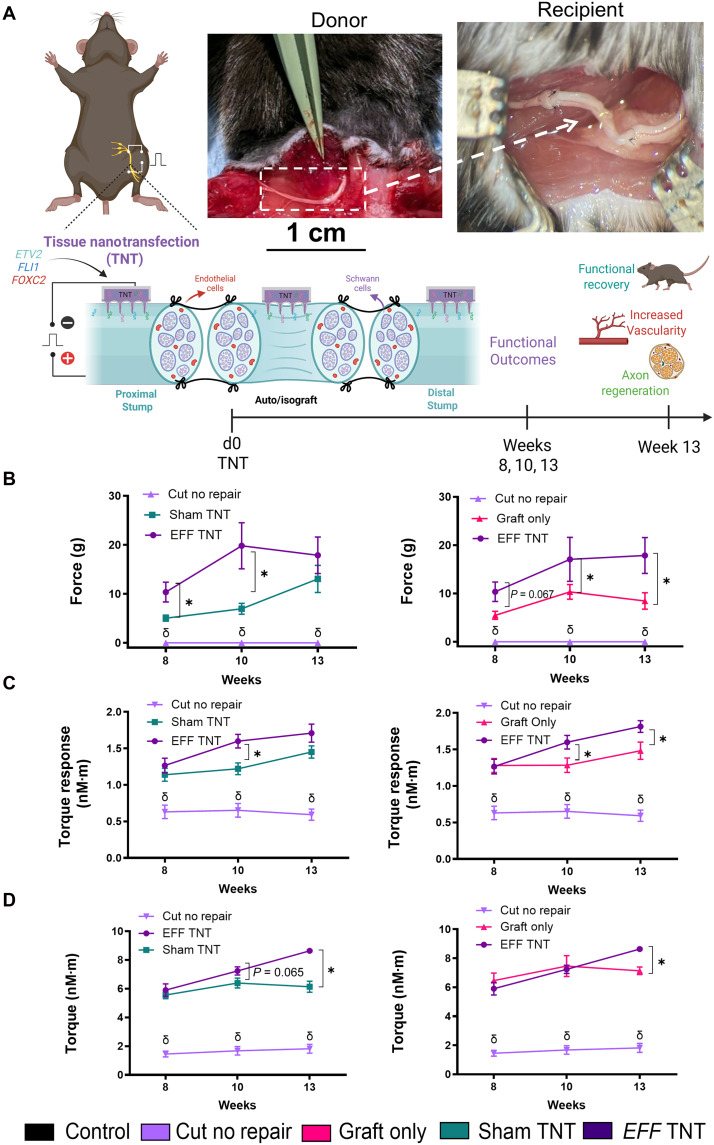
TNT with *EFF* improves functional recovery in sciatic nerve isografts. (**A**) Schematic representation and images illustrating vasculogenic TNT applied to isografts to induce vascular tissue formation, which guides axonal regeneration, followed by functional outcome measurements over a 13-week period. Segmental nerves (1-cm isografts) from donor mice were dissected, treated with TNT using *EFF* 1:1:2 or sham, and sutured into the nerve gap of genetically identical recipient mice. Before nerve grafting, TNT was applied to the proximal and distal stumps of the sciatic nerve in the recipient mice. Experimental groups included mice treated with sham or *EFF* 1:1:2 TNT, grafting without TNT (graft only), mice subjected to a 1-cm graft dissection without repair (cut no repair), and uninjured control mice. (**B**) Hindlimb grip strength significantly increased in the *EFF* TNT-treated group compared to the sham TNT and graft-only groups, initiating at week 8 and progressing through week 13. The cut no repair group failed to regain strength throughout the 13-week period. (**C**) In vivo muscle contractility assessment showed a significant increase in twitch torque in the *EFF* TNT group compared to sham TNT group at week 10 and compared to the graft only group at weeks 10 and 13. (**D**) Similarly, the *EFF* TNT group exhibited a significantly elevated tetanic torque compared to other groups at week 13, further suggesting enhanced muscle recovery and strength (*n* = 6 per group). All error bars are shown as SEM. # denotes significant difference with respect to the control, δ denotes significant difference with respect to cut no repair, and * denotes significant difference with respect to another group. δ and * with a *P* value < 0.05. Two-way ANOVA. Created in BioRender. A. Salazar (2026); https://BioRender.com/yn3s2jn.

Crush injury models are widely used to study nerve regeneration due to their reproducibility and ability to mimic aspects of axonal injury and repair (*34*). However, they do not fully replicate the severity or complexity of PNI typically observed in clinical settings, particularly in cases involving segmental nerve defects (*35*, *36*). Autologous nerve grafts remain the gold standard for repairing segmental nerve defects, as they provide essential structural support and a favorable microenvironment for regeneration (*37*). To investigate whether localized vasculogenic reprogramming via TNT can enhance nerve tissue repair by promoting vascularization that can support axonal growth, we used a sciatic nerve isograft model that mimics autografts used in the clinical practice. This model involved nerve grafts harvested from genetically identical donor mice. For these experiments, 8- to 10-week-old C57BL/6 mice served as both donors and recipients. A 1.3-cm segment of sciatic nerve was dissected from the donor mouse following TNT with the selected *EFF* 1:1:2 formulation. The graft was then measured, sized to 1 cm in length, and then grafted into the recipient mouse using microsurgical techniques. To further promote vascularization, TNT was also applied to the proximal and distal stumps of the recipient’s sciatic nerve prior to nerve grafting (Fig. 7A).

To better understand the kinetics of recovery following nerve injury and grafting procedures, we first conducted pilot studies in mice undergoing isografting, as previously described, but without TNT interventions. In these experiments, compound muscle action potential (CMAP) and motor unit number estimation (MUNE) were measured in response to sciatic nerve stimulation on a weekly basis over a 12-week period. Early postgrafting measurements revealed a marked reduction in CMAP amplitudes and MUNE in mice receiving isografts compared to uninjured controls, reflecting impaired signal transmission to the denervated muscle. Notably, CMAP and MUNE began to improve around weeks 6 to 7 and 7 to 8, respectively, with a gradual trend toward normalization by week 12 (fig. S6). Based on these findings, we determined that a 12- to 13-week period should be appropriate for evaluating long-term functional outcomes in our subsequent experiments combining grafting with TNT interventions. Given that meaningful functional changes started emerging between weeks 6 and 8 in the pilot study, we started outcome assessments at week 8 in the main study and extended the end point to week 13 to capture the later stages of recovery and ensure comprehensive evaluation of the regenerative response.

Based on the outcomes from this pilot study, we next evaluated functional recovery for over 13 weeks following TNT interventions (*EFF* 1:1:2 or sham) in combination with the grafting procedure. Functional outcomes included grip strength, muscle contractility, CMAP, and MUNE. The experimental groups also included mice treated with a graft without TNT (graft only), mice subjected to a 1-cm segmental defect without repair (cut no repair) and mice with no injury (control). Baseline measurements were collected for each mouse before surgery and subsequently evaluated at weeks 8, 10, and 13. Bilateral hindlimb grip strength, evaluated using a force transducer, revealed that the cut no repair group failed to regain strength throughout the 13 weeks, consistent with the absence of nerve continuity. In contrast, all other injury groups demonstrated a gradual recovery in grip strength. Notably, the *EFF* TNT-treated group displayed a significant increase in grip strength compared to the graft-only and sham TNT groups starting at week 8 and continuing through week 13 (Fig. 7B). To evaluate muscle contractile function, we measured peak torque production during twitch and tetanic plantar flexion contractions following sciatic nerve stimulation in vivo. Our findings demonstrated a significantly increased twitch torque in the *EFF* TNT group compared to sham TNT group at week 10 and compared to the graft only group at weeks 10 and 13, while the cut no repair group never showed improvements (Fig. 7C). Similarly, tetanic torque was significantly elevated in the *EFF* TNT group at week 13 compared to sham TNT and graft only groups (Fig. 7D), further indicating enhanced muscle recovery and strength, which was absent in the cut no repair group. We have also noticed a significant effect of time, with higher recovery at later time points. Significant differences were detected between week 8 and week 10, as well as between weeks 10 and 13, across all evaluated metrics (Fig. 7). When the data were analyzed by sex, we observed sex-associated variations in twitch torque in the *EFF* TNT group compared to sham TNT group at week 10 in male mice, whereas no differences were detected in female mice (fig. S7A). Likewise, tetanic torque was significantly higher in the *EFF* TNT group at week 13 compared to the graft only and sham TNT groups in male mice, whereas in female mice, only differences were found in the *EFF* TNT compared to the sham TNT group at week 13 (fig. S7B). These differences highlight potential sex-dependent responses that should be explore in future studies.

Last, CMAP amplitudes and MUNE were assessed to determine nerve signal transmission and the number of functional motor units in the injured groups. At early time points, all injured groups showed significant reductions in CMAP amplitude and motor unit number compared to the positive control, reflecting the initial disruption of nerve signaling. Over time, however, CMAP amplitudes and motor unit numbers gradually improved in the graft-only, sham TNT, and *EFF* TNT groups compared to cut no repair. While recovery trends were observed in all groups, there were no significant differences between the TNT-treated groups and the graft only group by week 13 (fig. S8, A and B).

When normalized to clinically relevant benchmarks commonly used in human PNI repair, our mouse isograft model demonstrated 72% recovery in twitch force, 74% in tetanic force, and 62% recovery in CMAP relative to the uninjured group at 13 weeks postintervention, placing the TNT-*EFF* intervention within the range associated with clinically “good” motor recovery (*38*, *39*). These findings demonstrate that integrating vasculogenic TNT with nerve grafting procedures can significantly enhance functional recovery in segmental nerve defects, underscoring its potential as a clinically translatable strategy to improve outcomes in severe PNI.

**Fig. 8.**
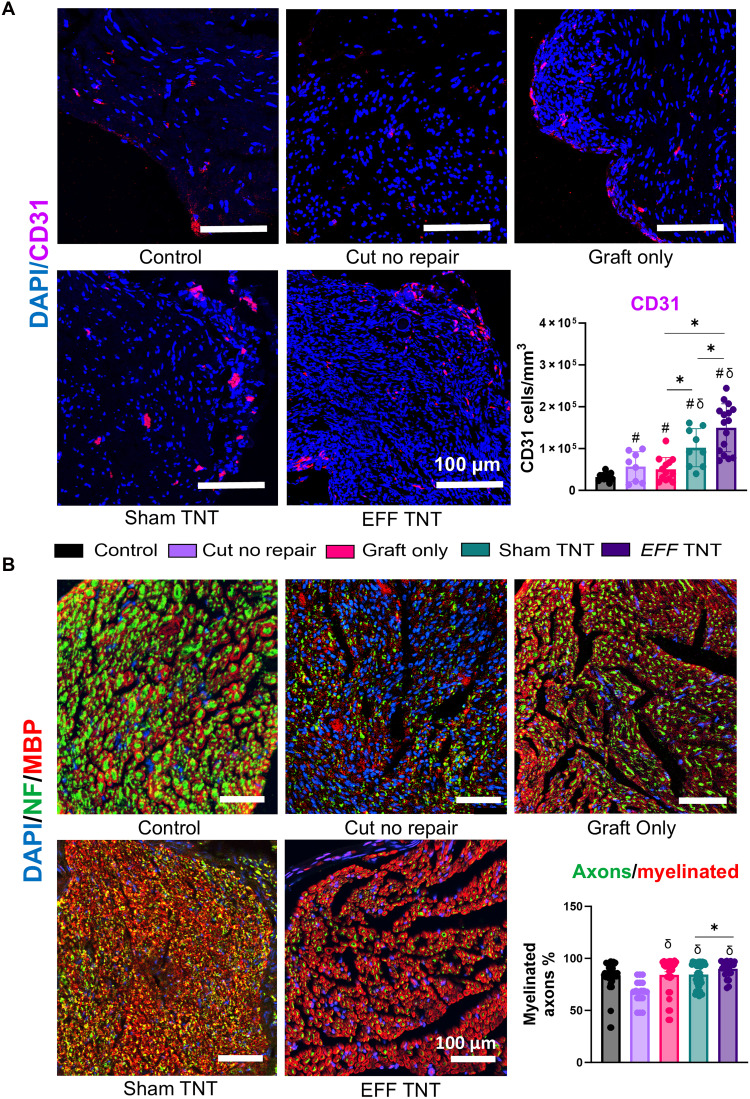
TNT with *EFF* improves vascular tissue formation and axon regeneration in sciatic nerve isografts after 13 weeks. Representative images and histological analysis of the isografts 13 weeks post grafting and TNT intervention reveal (**A**) an overexpression of the vascular marker CD31 (purple), colocalized with DAPI (nuclei marker, blue), in the *EFF* and sham TNT groups compared to control groups, with a significant difference between the *EFF* TNT and sham TNT groups. (**B**) Axon regeneration and functionality were assessed by the expression of the axonal marker (NF, green) and myelination by the expression of MBP (red). Analysis of the percentage of myelinated axons showed a higher proportion of axons (green) that were also positive for MBP (red) in the *EFF* TNT group compared to sham TNT group (*n* = 6 per group). All error bars are shown as SEM. # denotes significant difference with respect to the control, δ denotes significant difference with respect to cut no repair, and * denotes significant difference with respect to another group. #, δ, and * with a *P* value < 0.05, one-way ANOVA.

After 13 weeks postgrafting and TNT intervention, the gastrocnemius and soleus muscles from both the injured (right) and contralateral hindlimb (left) were collected and weighed. In addition, sections (1 cm) of the sciatic nerve were collected for histological analysis, with uninjured contralateral sciatic nerves serving as baseline controls. Results revealed a significant reduction in muscle weight of the gastrocnemius from the injured limb compared to the control. Although the muscle weights in the graft only, sham TNT, and *EFF* TNT groups remained significantly lower than the uninjured control, they showed signs of recovery, approaching normal levels over time, as opposed to the cut no repair group (fig. S8C). In contrast, the contralateral (left) gastrocnemius and soleus muscles displayed no significant differences among the groups (fig. S8D). The histological analysis of the sciatic nerve sections revealed a significant increase in CD31^+^ cells in the *EFF* TNT and sham TNT groups compared to controls and a significant difference between the *EFF* TNT and sham TNT groups (Fig. 8A). Further evaluation of axonal regeneration using NF and myelin basic protein (MBP) markers showed a higher percentage of myelinated axons in the *EFF* TNT group compared to sham TNT (Fig. 8B). These findings reveal that localized vasculogenic signaling via TNT not only improves functional recovery but also enhances graft vascularization and myelinated axon regeneration, positioning this strategy as a clinically relevant approach to advance outcomes in segmental nerve repair.

To investigate the early effects of vasculogenic TNT on the formation of blood vessels within the graft, we first performed quantitative histological analyses of CD31 expression in longitudinal nerve sections at 7 days postgrafting. These analyses revealed a significant increase in blood vessel formation in the middle portion of the graft treated with *EFF* TNT compared to sham TNT (fig. S9A). These results suggest that vasculogenic TNT supports early formation of blood vessels, a process that may be critical for creating a proregenerative environment that supports axonal growth and enhances functional recovery in surgically reconstructed segmental nerve defects. To further investigate the structural organization of regenerating tissue at an intermediate stage, we examined longitudinal nerve sections at 5 weeks postgrafting. Notably, axons were closely coaligned with CD31^+^ blood vessels, suggesting that the early vascular networks induced by vasculogenic TNT may serve as a supportive and organized scaffold for axonal regeneration (*40*). This aligned architecture closely resembled that of healthy nerve tissue, indicating that the *EFF*-TNT treatment contributes not only to vascularization but also to meaningful structural repair (fig. S10A).

### RNA-seq reveals transcriptional changes associated with vasculogenic reprogramming in nerve grafts

**Fig. 9.**
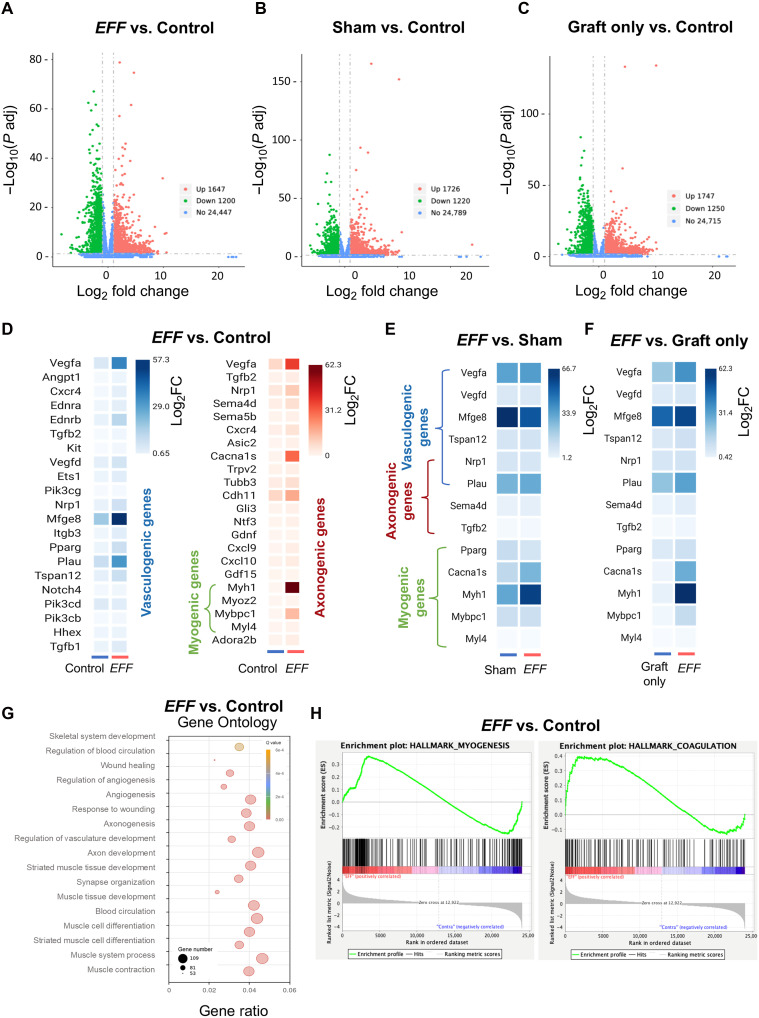
Transcriptomic analysis reveals the impact of BPs underlying the enhanced functional recovery and vascular tissue formation of TNT following isografts. RNA-seq analysis was performed on sciatic nerve tissue 13 weeks after TNT applied to isografts comparing the injured groups *EFF* TNT, sham TNT, and graft only to healthy tissue (control). Volcano plots displaying the number of up-regulated and down-regulated genes in the (**A**) *EFF* (**B**) sham-TNT and (**C**) graft only compared to control. Up-regulated genes are shown in red, down-regulated in green, and blue represents non-DEGs. (**D**) Clustered heatmaps show the expression patterns of vasculogenic and axonogenic-associated DEGs between *EFF* TNT–treaded isografts compared to control. Similarly, subtle transcriptional differences in the expression of vasculogenic, axonogenic, and myogenic genes were identified when comparing the *EFF* group with (**E**) sham TNT or (**F**) graft only. (**G**) GO enrichment analysis reveals the BP related to angiogenesis, vascular development, axonogenesis, axon development, and muscle tissue development in the *EFF* group compared to healthy tissue. (**H**) Last, a GSEA showing the significant activation of the myogenesis and coagulation pathways in the *EFF* group compared to the control. Significantly DEGs were considered with Log_2_FC ≥ ∣2∣ and adjusted *P* value ≤ 0.05.

To investigate the BPs underlying enhanced functional recovery and vascular tissue formation observed with the *EFF* treatment, we examined the transcriptome of the graft after 13 weeks postintervention across the five experimental groups (fig. S11A) (*n* ≥ 3). Total RNA from the nerve graft was isolated and sequenced. Transcriptomic analysis revealed that grafting and TNT interventions led to transcriptional shifts compared to healthy nerve tissue. The *EFF* TNT group exhibited 1647 up-regulated and 1200 down-regulated genes relative to control (Fig. 9A). Similarly, 1726 up-regulated and 1220 down-regulated genes were identified in the sham TNT group (Fig. 9B), 1747 up-regulated and 1250 down-regulated genes in the graft only group (Fig. 9C), and 1632 up-regulated and 860 down-regulated genes in the cut no repair group compared to the healthy control (fig. S11B) and to the *EFF* TNT-treated grafts (fig. S11C). Unsupervised hierarchical clustering of all expressed genes revealed distinct transcriptional profiles between the *EFF*-treated and control groups (fig. S11D). Heatmap clustering showed distinct expression patterns of vasculogenic-, axonogenic-, and myogenic-associated DEGs in *EFF*-treated samples compared to the healthy control. Notably, key genes (*Vegfa*, *Ednrb*, *Nrp1*, *Mfge8*, *Pparg*, *Plau*, *Cacna1s*, *Cdh11*, *Myh1*, and *Mybpc1*), relevant to angiogenesis, endothelial cell migration, axonal growth and repair, and myogenesis were enriched in the *EFF* TNT group relative to healthy tissue, supporting the observed histological and functional enhancements (Fig. 9D and fig. S11E). While direct comparisons between *EFF* TNT and sham TNT/graft only groups revealed more limited transcriptional differences, an up-regulation of *Vegfa*, *Mfge8*, *Plau*, *Cacna1s*, *Myh1*, and *Mybpc1* were observed in *EFF*-treated nerves compared to sham (Fig. 9E) and graft only (Fig. 9F). These genes are associated with vascular remodeling, endothelial migration, axonal repair, and muscle function. GO enrichment analysis confirmed that DEGs in the *EFF* group were significantly associated with BP related to angiogenesis, vascular development, axonogenesis, axon development, and muscle tissue development (Fig. 9G) compared to the control. Similarly, GSEA revealed enrichment of processes and signaling pathways involved in angiogenesis, neuroprotection, endothelial and axon regeneration, cellular plasticity, and myogenesis in the *EFF* group compared to healthy tissue (Fig. 9H and fig. S11F). Pathways such as myogenesis, coagulation, IL-6 Jak Stat3, angiogenesis, and complement activation were significantly up-regulated, reinforcing the biological relevance of these transcriptional changes in driving functional recovery. Although RNA-seq at week 13 revealed more limited transcriptional differences between the *EFF* and sham/graft groups, the data suggest that early activation of vascular and neuronal repair pathways may have resulted in the superior functional recovery observed in *EFF*-treated animals (Fig. 9, E and F). The sustained up-regulation of vasculogenic, axonogenic, and myogenic programs correlates well with enhanced muscle contractility, axonal function, and vascularization, indicating that these early transcriptional shifts likely contributed to the long-term regenerative benefits conferred by *EFF*-TNT.

## DISCUSSION

Our results, together with previous studies (*22*, *23*, *41*, *42*), establish TNT as a powerful, nonviral nanotechnology platform for direct in vivo gene delivery, supporting therapeutic applications in cell transdifferentiation across a range of conditions. TNT delivers genetic cargo through nanochannels directly into tissues in a controlled, voltage-dependent, and minimally invasive manner, preserving tissue cytoarchitecture and avoiding structural damage. In this study, we demonstrate for the first time the ability of TNT to induce controlled vasculogenic reprogramming within nerve grafts following reconstruction of a segmental nerve defect, promoting the formation of new vasculature, supporting axonal growth, and enhancing functional recovery. These findings position TNT as a promising strategy capable of accelerating nerve repair and improving outcomes in peripheral nerve reconstruction, offering a nonviral, tissue-regenerating alternative for therapeutic gene delivery in complex injury environments.

The close synergy between vasculogenic and neurogenic processes during tissue repair and development has made vasculogenic therapies an attractive approach for neurodegenerative diseases, as newly formed blood vessels provide neurotrophic support for nerve tissue repair and regeneration (*10*). In this context, direct transdifferentiation holds a substantial promise for developing patient-specific autologous therapies, addressing challenges of cell scarcity and functional limitations associated with traditional autologous approaches. This groundbreaking strategy uses lineage-specific TFs to convert one cell type into another by overcoming the cells’ existing epigenetic fate and resetting it to generate the desired cell type. Cell reprogramming has already demonstrated potential in disease modeling and therapeutic development (*43*). Notably, direct transdifferentiation overcomes major limitations faced by traditional cell therapies by using readily available cell sources (e.g., fibroblasts) and offering a more controlled and potentially safer alternative by bypassing the pluripotent stage, thereby reducing the risk of tumor formation associated with iPSC-based methods (*44*). Although the expression of reprogramming TFs is often transient, our group (*24*) and others (*45*, *46*) indicate that the induced reprogrammed cells can remain stable for several weeks, likely due to epigenetic reinforcement of the new linage state, yet future studies are needed to verify the long-term stability of the acquired phenotype (*44*).

The *EFF* cocktail used in this study has been developed and previously implemented by our group to drive provasculogenic conversions in somatic cells (e.g., fibroblasts) for fundamental and translational applications (*22*–*25*), whereas other groups have extensively used Etv2 alone or in combination with different factors to promote this conversion (*47*–*49*). The selection of TFs is typically informed by developmental biology insights into cell lineage specification. Among these, members of the ETS (E26 transformation-specific) TF family play crucial roles in regulating cell differentiation, proliferation, survival, and angiogenesis, through their conserved ETS domain, which binds to GGAA/T DNA sequences to control gene expression. *Etv2*, a pioneer ETS family member, governs endothelial cell fate by activating key vascular genes essential for blood vessel formation, playing a pivotal role in vascular development during embryogenesis with transient expression between embryonic days 7 and 9.5 (*19*). *Fli1*, another ETS factor, regulates endothelial survival and vascular development by modulating angiogenesis-related genes. Its expression is initially triggered by *Etv2* during early development, after which *Fli1* sustains its own expression and modulates *Etv2*-dependent endothelial genes critical for vascular morphogenesis and homeostasis (*20*). Complementing these, *Foxc2* is a key transcriptional regulator in VEGF-mediated vascular formation and remodeling under both physiological and pathological conditions. *Foxc2*, once activated by VEGFA, binds to the promoter region of delta-like canonical Notch ligand 4 (Dll4), up-regulating its expression and subsequently initiating Notch signaling (*21*). Clinically, VEGF plays an important role in shaping vasculogenic reprogramming, as this pathway supports endothelial cell identity and activates functional angiogenic programs, contributes to blood vessel formation, and promotes the proliferation and survival of endothelial cells and Schwann cells (*50*, *51*). Together, *EFF* form a synergistic transcriptional network that can effectively induce the conversion of somatic cells into endothelial cells by recapitulating essential vascular developmental programs.

Our study aimed to investigate how varying the mass ratios of these TFs would affect reprogramming outcomes. To this end, we generated seven different *EFF* formulations by doubling the expression of each TF individually or in combination. We found that doubling *Foxc2* expression significantly enhanced reprogramming efficiency, leading to the up-regulation of vasculogenic-related genes both in vitro and in vivo while promoting vascular tissue formation in a peripheral nerve crush injury model. We hypothesize that the differences observed at 7 days in vitro reflect the hierarchical roles of the TFs, with higher *Foxc2* expression crossing the threshold required to activate downstream vasculogenic programs that are not fully engaged in other formulations. This enhanced reprogramming outcome is consistent with the pivotal role of *Foxc2* in regulating vascular formation through VEGF-mediated Notch signaling. In in vivo experiments, RNA-seq analysis from tissues treated with the 1:1:2 *EFF* formulation revealed significantly increased expression of key vascular genes, including *Notch4* (endothelial cell signaling), *Pik3cd, Pik3cb* (angiogenic survival pathways), *Hhex* (vascular development and differentiation), *Tgfb1* (vessel stabilization and remodeling), and *Esm1* (endothelial sprouting and vascular permeability). Notably, the 1:1:2 *EFF* formulation led to a CD31 overexpression in vivo, which may reflect the stronger influence of the tissue microenvironment, inflammatory signaling, and endogenous angiogenic factors that may selectively amplify *Foxc2*-dependent pathways (*52*).

In addition, RNA-seq from in vitro experiments at 24 hours posttransfection revealed elevated expression of *Foxc2*, as expected. Moreover, important regulators such as *Notch2* and *Notch4* (involved in endothelial cell fate and vessel maturation) (*53*) and *Dll1* and *Dll4* (key ligands activating Notch signaling for endothelial sprouting and migration) (*54*), along with *Nrp1* and *S1pr1* (associated with endothelial cell migration, vessel stabilization, and angiogenic signaling), were up-regulated. The significant enrichment of BP related to angiogenesis, vasculogenesis, and blood vessel formation were already detected at this early time point. Notably, these transcriptional changes emerged long before any morphological or functional conversion occurred, highlighting a rapid and robust transcriptional response preceding full cellular reprogramming. The observed up-regulation of *Notch2*, *Notch4*, *Dll1*, and *Dll4* in the *EFF* 1:1:2 group supports our hypothesis that suggest that increased *Foxc2* levels enhances reprogramming through Notch-related and VEGF-responsive pathways, reinforcing endothelial identity and promoting the expression of downstream effectors needed for vascular remodeling, ultimately improving reprogramming efficiency.

A potential concern with applying reprogramming therapies within injured peripheral nerves is the risk of disrupting the essential repair functions of Schwann cells, which adopt a regenerative phenotype crucial for axonal regeneration. A major risk would be depleting these cells through their unintended conversion into vascular tissue, impairing nerve regeneration. However, our data indicate that fibroblasts are the primary cell type undergoing vasculogenic reprogramming, as reflected by a higher percentage of CD31^+^GFP^+^ cells derived from fibroblasts compared to Schwann cells. A baseline level of GFP-CD31 coassociation was detected in control nerves. For fibroblasts, this is consistent with reports describing their capacity for mesenchymal-to-endothelial transitions (*55*), potentially explaining CD31 expression in untreated nerves. In Schwann cells, although they are not expected to spontaneously express CD31, their close association with endothelial cells and secretion of angiogenic factors following injury (*56*) could account for this signal in sham-treated nerves. Notably, our findings align with the expected cellular composition of the nerve, with GFP-CD31 double-positive cells comprising 30% in s100B (Schwann cell mice) and 10 to 20% in s100A4 (fibroblast mice), aligning with reported distributions of ~29.6% Schwann cells and 20.6% fibroblasts in peripheral nerve tissue (*57*). Although off-target effects remain theoretically possible and a small subset of Schwann cells residing in the epineurium could be transfected, potentially influencing Schwann cell biology or repair-associated programs (*58*), our data indicate that *EFF*-mediated reprogramming is restricted to fibroblasts and does not induce endothelial conversion in Schwann cells. This apparent cell type–specific responsiveness substantially mitigates the risk of disrupting Schwann cell–driven regenerative processes. Moreover, because TNT-mediated *EFF* expression is episomal and transient, the likelihood of long-term or irreversible alterations to Schwann cell phenotype or function is further reduced. These observations together with our prior in vitro findings demonstrating greater vasculogenic plasticity in fibroblasts compared to Schwann cells (*23*) reinforce that fibroblasts are the main contributors to the newly formed endothelial population in both settings in vitro and in vivo, alleviating concerns about Schwann cell depletion compromising nerve repair.

The crush injury model is a simple, widely used and validated approach to study peripheral nerve repair. It simulates axonotmesis, producing controlled and reproducible injuries while preserving the nerve’s structural integrity, allowing for spontaneous axonal regeneration without surgical repair. In previous work using this model, we also evaluated TNT-mediated *EFF* delivery and confirmed that it did not impair nerve function (*23*), and although this model has provided valuable insights into axon degeneration, Schwann cell responses, and Wallerian degeneration, it has substantial limitations. It overestimates the regenerative capacity of peripheral nerves since the connective tissue and nerve continuity remain intact, and it fails to replicate segmental nerve defects often encountered in clinical settings (*59*). PNI with segmental nerve defects cannot be repaired using tensionless primary neurorrhaphy and require grafting strategies such as autografts, allografts, and conduits. Autograft remains the gold standard, offering Schwann cells, endothelial cells, and a supportive microenvironment, making them the most effective option for promoting regeneration. However, they are limited by donor site morbidity, graft availability, and less than ideal functional outcomes (*60*). To address these challenges, the most promising strategy lies in enhancing autografts using tissue engineering and gene-based therapies, combining their inherent advantages with regenerative cues to improve outcomes in peripheral nerve repair.

We demonstrated that a single intervention of vasculogenic reprogramming through TNT applied to grafts is sufficient to drive remarkable functional recovery, restoring both force generation and muscle contractility following PNI. In bilateral hindlimb grip strength assessment, the cut no repair group failed to regain force throughout the 13-week period, while all other injury groups exhibited progressive recovery. Notably, the *EFF* TNT-treated group displayed a significant improvement in grip strength compared to grafting controls starting at 8 weeks postinjury. To further assess functional muscle recovery following nerve damage, we measured twitch and tetanic plantar flexion contractions. The *EFF* TNT group demonstrated a significant increase in twitch torque relative to other groups, reflecting the muscle’s immediate response to a single nerve-muscle stimulus, which is an early indicator of reestablished nerve-muscle communication. Similarly, tetanic torque—which measures the muscle’s ability to sustain a strong, continuous contraction under high-frequency stimulation—was significantly higher in the *EFF* TNT group. This indicates not only restored connectivity but improved muscle strength and endurance, supporting a more complete and sustained functional recovery. Together, these findings highlight the profound regenerative impact of a single, nonviral gene delivery intervention. Clinically, this strategy would translate to an intraoperative workflow in which TNT is applied directly to the graft immediately before transplantation. As with other regenerative approaches, patient factors such as age and sex may influence outcomes. For example, aging is associated with reduced cellular plasticity (*61*) and slower nerve regeneration (*62*). These considerations suggest that the effectiveness of TNT-mediated reprogramming may vary with age and sex and should be explored further.

To investigate the mechanisms underlying the functional recovery observed in the treatment group, we examined vascularization and axon regeneration in the nerve tissue at different time points. At 13 weeks postgrafting and TNT intervention, we found a significant increase in CD31^+^ cells in both the *EFF* TNT and sham TNT groups compared to controls, with *EFF* TNT showing a higher vascular density than sham TNT. The analysis of axonal regeneration revealed a greater proportion of axons coassociated with MBP in the *EFF* TNT group, indicating enhanced myelination and axon regeneration. To assess early vascular effects, we evaluated blood vessel formation within the graft and observed a significant increase in vascularization in the middle region of the graft of *EFF*-TNT–treated nerves compared to the sham-TNT group, suggesting that early induction of vasculature through vasculogenic reprogramming was likely key in supporting subsequent regeneration and functional recovery. As expected, by 13 weeks, vascularization increased across all injury groups, possibly due to the natural inflammatory response to nerve injury, which is essential for activating repair mechanisms (*40*). Notably, the sham-TNT group also exhibited greater vascularization than the graft-only and cut no repair groups, possibly attributable to the fact that nerve injury alone induces inflammation, while plasmid DNA delivery further amplifies inflammatory and regenerative signals by transiently activating immune pathways (*63*). This inflammatory response is expected to be transient and may help, rather than impair, regenerative reprogramming (*64*, *65*). This synergy may contribute to the increased vascularity seen across TNT groups. However, the distinct advantage of the *EFF*-TNT group lies in its ability to directly induce vasculogenic reprogramming, which likely accounts for the superior vascularization, enhanced axonal regeneration, and ultimately better functional outcomes compared to sham-TNT. Last, another possible explanation can be due to the effects of the electrical stimulation applied during TNT, as electric fields have been shown to promote axonal and muscle reinnervation (*66*).

In both our in vitro and in vivo RNA-seq analyses, we systemically examined the enrichment of up-regulated pathways in our lead *EFF* 1:1:2 formulation compared to controls. We identified activation of several key pathways, including angiogenesis, EMT, complement activation, IL-6/Jak/Stat3, PI3K/AKT/mTOR signaling, coagulation, and myogenic pathways. These pathways are associated with processes relevant to our therapeutic goals, such as angiogenesis, cellular plasticity, neuroprotection, endothelial and axon regeneration, and muscle preservation. Upon further investigation, we found that EMT-related pathways, by promoting cellular plasticity, may facilitate endothelial reprogramming and support neuronal repair (*67*). Complement activation contributes to neuroprotection and axon regeneration through factors such as C1q and C3a, which help facilitate the recruitment and activation of immune cells (*68*). The IL-6/Jak/Stat3 pathway plays a direct role in modulating Schwann cell behavior, inflammation, and axonal regeneration. IL-6 functions as a neurotrophic factor in peripheral nerve regeneration (*69*), while STAT3 is up-regulated in regenerating neurons following axonal injury (*70*). Last, PIK3/AKT/mTOR pathway is well-established as essential for endothelial cell survival, proliferation, and angiogenesis (*71*). Together, these findings reinforce the successful role of vasculogenic reprogramming in promoting blood vessel formation, which in turn supports axonal regeneration and nerve repair.

We developed and applied a TNT-based gene delivery approach to enhance peripheral nerve regeneration by inducing vasculogenic reprogramming within nerve grafts. Our results demonstrate that optimizing a vasculogenic cocktail significantly improved reprogramming efficiency, up-regulating vasculogenic-related genes in vitro and in vivo, and promoting vascular tissue formation in nerve crush injury models. Lineage tracing further identified fibroblasts, rather than Schwann cells, as the predominant contributors to the newly formed vasculogenic cell population following TNT. This study represents the first application of vasculogenic TNT in combination with surgical nerve graft reconstruction to address complex segmental nerve defects. This combinatorial approach resulted in increased blood vessel density and a higher number of myelinated axons within the graft, ultimately leading to improved functional recovery, including enhanced grip strength and muscle contractility observed at 13 weeks postsurgery. Histological and transcriptional analyses corroborated the activation of vasculogenic, axonogenic, and myogenic pathways, supporting the structural and functional restoration of the injured nerve. Together, these results establish TNT as a promising, nonviral platform for locally inducing therapeutic vasculogenesis and promoting tissue repair, offering a previously unidentified strategy to improve regenerative outcomes in peripheral nerve reconstruction.

## MATERIALS AND METHODS

### DNA plasmid preparation

All the plasmids were purchased from Origene Technologies, and the corresponding catalog numbers are listed in table S1. After bacterial transformation, the plasmids were isolated using the ZymoPURE II Plasmid Midiprep Kit (Zymo Research) according to the protocol provided by the manufacturer. The plasmid concentrations were measured with NanoDrop 2000 (Thermo Fisher Scientific).

### In vitro cell culture and nonviral transfection

Fibroblasts were purchased from EMD MilliporeSigma (PMEF-HL) and cultured in Dulbecco’s modified Eagle’s medium (Thermo Fisher Scientific) supplemented with 10% fetal bovine serum (VWR) and 1% penicillin/streptomycin (Gibco) at 37°C in a humidified air chamber containing 5% CO_2_. Once the cells reached a confluency level greater than 80%, they were detached, and 1.0 × 10^6^ cells were resuspended in 100 μl of electrolytic buffer for subsequent nonviral cell transfection using a Neon transfection system (Thermo Fisher Scientific). Cells were cotransfected with three plasmid vectors encoding for Etv2, Fli1, and Foxc2 at 0.05 to 0.1 μg/μl, depending on the *EFF* formulation (table S2). pCMV6 (sham/empty vector) was used as a control with the same final gene concentration (0.15 to 0.2 μg/μl). Following the procedure assigned by the manufacturer (Invitrogen: Neon Transfection System), the cells were transfected for 30 ms with 1 pulse at 1425 V. After transfection, the cells were seeded and maintained in culture for 24 hours or 7 days depending on the experiment. The transfection efficiency of the molecular cargo inside the cells was inspected by quantitative reverse transcription polymerase chain reaction (qRT-PCR) for each factor and immunofluorescence microscopy (GFP-tag positive signal).

### Gene expression analyses

Total RNA was isolated from the cells with TRIzol reagent (Invitrogen) following the protocol recommended by the manufacturer. Isolated RNA was treated with deoxyribonuclease (DNase) (Thermo Fisher Scientific) to eliminate the presence of coprecipitated extracellular DNA. The samples were then quantified via spectrophotometry using the NanoDrop 2000 (Thermo Fisher Scientific), and RT reactions were performed using the Maxima First Strand cDNA Synthesis Kit (Thermo Fisher Scientific) from 500 to 2500 ng of RNA in a 20-μl reaction, maintaining the same amount of cDNA for all the samples. Taking cDNA as the template, mRNA expression levels were validated with qRT-PCR using TaqMan fast advance Master Mix (Thermo Fisher Scientific) and predesigned primers (table S3). Glyceraldehyde-3-phosphate dehydrogenase was used as a normalizing gene in all qRT-PCR.

### RNA sequencing

Total RNA from sciatic nerve tissue was collected in TRIzol reagent and homogenized using poly-T oligo-attached magnetic beads. RNA was then isolated using the PureLink RNA Mini Kit (Invitrogen), followed by DNAse treatment and cDNA synthesis as previously described. Total RNA quality was assessed using the Agilent 2100 Bioanalyzer (Agilent Technologies) with the RNA 6000 NanoAssay kit by Chip, and samples with an RNA integrity number (RIN) ≥ 6 were sent for sequencing. RNA-seq was performed and analyzed by Novogene Co., following their standard pipeline. RNA-seq libraries were prepared with end repair, A-tailing, adapter ligation, USER digestion, size selection, PCR amplification, and purification. Library quality and concentration were assessed using Qubit, real-time PCR, and a Bioanalyzer. Libraries were pooled and sequenced on Illumina platforms, generating FASTQ files. Reads were quality-filtered to remove low-quality and adapter-containing sequences. Clean reads were mapped to the mouse genome using Hisat2 (v2.0.5), and gene expression was quantified with featureCounts (v1.5.0-p3) and expressed as FPKM. Differential expression analysis was performed using DESeq2 (v1.20.0), with *P* values adjusted by the Benjamini-Hochberg method [false discovery rate (FDR) ≤ 0.05] and a fold change cutoff of 2. GO enrichment was conducted using clusterProfiler. For each pair-wise comparison, DEGs were identified and illustrated by a volcano plot, a GO analysis with the BPs overrepresented in the listed genes, a heatmap clustering, and a subsequent GSEA. All figures were generated using Novomagic, the analysis platform provided by the sequencing company. GSEA was performed using GSEA software (Broad Institute, UC San Diego) with MSigDB gene sets. Only gene sets with FDR < 25% were considered significant. Results were visualized using GSEA’s built-in plotting functions. The bulk RNA-seq data are available through the Gene Expression Omnibus (GEO) with the accession codes: GSE317915 (in vitro RNA-seq), GSE319328 (7 day in vivo RNA-seq), and GSE319284 (13 weeks in vivo RNA-seq).

### TNT chip fabrication and simulation

TNT chips were fabricated using 4-inch, 200-μm-thick, double-sided polished silicon wafers in a Class 100 cleanroom. TNT chips have nanochannels and needles on the front side connecting to the microchannels on the backside. Wafers were first vapor-primed with hexamethyldisilazane to improve photoresist adhesion before spin-coating with AZ1512-positive photoresist (~1-μm thick). Nanochannels (~0.8- to 1-μm diameter, 12- to 15-μm depth) were patterned using direct-write lithography and etched via deep reactive ion etching. The photoresist was then stripped with acetone. Needles were fabricated on the nanochannel side by patterning and etching microscale pillars. On the wafer’s backside, microchannels (~150-μm diameter) were patterned, exposed using lithography, and etched until they connected with the nanochannels. Wafers were then coated with a 50-nm silicon nitride insulating layer using chemical vapor deposition. The devices were also characterized using SEM images of the cross section to ensure quality.

Simulations were performed in Google Colab using a custom Python-based model to replicate the physical and electrical behavior of a TNT chip. The simulation of the chip geometry included microchannels (150-μm wide, 185-μm deep) and nanochannels (0.9-μm wide, 11-μm deep) on a silicon substrate, with conical nanostructures (4-μm base, 4.5-μm height) to enhance electric field localization. A 200 V was applied for 10 ms, with 65% of channels open. Murine sciatic nerve electrophysiology was modeled using the following parameters: resting potential (−70 mV), action potential threshold (−55 mV), nanoporation threshold (−200 mV), refractory period (1 ms), conduction velocity (120 m/s), and sodium channel density (600 channels/μm^2^). Membrane impedance was considered extremely high, approaching infinity, consistent with literature reports for peripheral nerves such as the sciatic, where tight junctions and myelin limit passive current flow (*72*). A 100 × 100 spatial grid was used to simulate the electric field, applying Gaussian smoothing. The lower triangular domain represented sciatic nerve tissue, where nanoporation sites were defined by fields exceeding threshold. Biological response was modeled with a randomized Gaussian distribution to simulate variable transfection efficiency. Figures were generated using matplotlib, numpy, scipy.ndimage, and mpl_toolkits.axes_grid1.

### Animal husbandry

All experiments received approval from the Institutional Animal Care and Use Committee of The Ohio State University (IACUC protocol #2016A00000074-R1). For linage tracing experiments, transgenic mouse models B6;D2-Tg(S100B-EGFP)1Wjt/l and B6.Cg-Tg(S100a4-EGFP)M1Egn/YunkJ (the Jackson Laboratory) were used to trace different cell populations in the sciatic nerve, expressing GFP in Schwann cells and fibroblasts, respectively. Mice were 7 to 10 weeks old, and only males were used. For all other in vivo experiments, 7- to 10-week-old male and female C57BL/6J mice (the Jackson Laboratory) were used. For all studies, the animals were housed no more than five animals per cage in a sterile barrier, controlled vivarium with a 12-hour light/12-hour dark cycle, and had unrestricted access to food and water. All animals were acclimated to new housing conditions for 1 week before behavioral testing (e.g., grip, Ephys, and Mphys) and operation. Before surgery, mice were injected with Buprenorphine ER (extended-release opioid) at 0.1 mg/kg (animal weight) for pain management postoperative (post-op). During surgery, 5% isoflurane and atmospheric air was used to anesthetize the animals and 1.2 to 2% isoflurane to maintain them during the surgical procedure (10 to 40 min per mouse). Mice were shaved in the surgical area (right leg) and sterilized with 70% isopropyl alcohol and betadine (or chlorohexidine) three times.

For the crush injury model, a 1-cm right unilateral incision was made on the skin on the medial-lateral portion of the limb just below the hip joint to expose the sciatic nerve, followed by its separation from the surrounding tissue and fascia. The crush injury was done by using 1-mm-wide locking hemostatic forceps two times for 15 s with a 15-s release in between. TNT was then performed on the sciatic nerves with either the *EFF* treatment or the empty vector pCMV6 (sham) as previously described (*22*, *23*). Briefly, the TNT reservoir was filled with 350 μl of the plasmid solution containing a concentration of 0.15 to 0.2 μg/μl (depending on the formulation) for the plasmids. A positive electrode is connected to a 25-gauge needle positioned under the sciatic nerve, and a negative electrode iconnected to a gold electrode (Neon Electroporation System, Thermo Fisher Scientific) filled with a 0.9% sodium chloride saline solution is placed inside the plasmid reservoir. The TNT chip was then placed on top of the sciatic nerve, and a total of 200 V, 100 ms, and 10 pulses were used to deliver the respective plasmid treatment into the injured sciatic nerve. Upon completion of the transfection, the incision wound was closed using interrupted nylon sutures, and mice were individually housed with mash and hydrogel-supporting diet, placed on a heating pad (37°C). Mice were monitored daily for 5 days post-op to ensure proper recovery.

For the isograft model, 7- to 10-week-old C57BL/6 mice served as both donors and recipients. The sciatic nerve tissue from the donor mice was exposed and treated with TNT as previously described, and a 1.3-cm segment of sciatic nerve was measured and dissected from the level of the exiting nerve root to just beyond the sciatic trifurcation. The right sciatic nerve of the recipient mice was exposed, treated with TNT, and then transected 3-mm proximal to the distal trifurcation. The harvested nerve graft was measured and cut to 1 cm and then sutured into the nerve gap. The distal end of the donor graft was positioned facing the proximal nerve stump of the recipient using 10-0 nylon microsutures, secured with loose knots to allow optimal regeneration. TNT was also applied to the proximal and distal stumps of the recipient’s sciatic nerve before grafting. The experimental groups included mice treated with grafting without TNT (graft only) as the graft control, mice subjected to a 1-cm graft dissection without repair (cut no repair) as the negative control, and mice with no injury (control) as the positive control.

### Functional outcomes

For the functional outcomes, baseline measurements were collected for each mouse before surgery and subsequently evaluated at weeks 6, 8, 10, and 13 weeks. For each testing session, animals were weighed at the beginning of the measurements. Functional outcomes included twitch and tetanic plantar flexion contraction torque, grip strength and electrophysiological measures of CMAP amplitude, and average single motor unit potential (SMUP) amplitude, from which MUNE was calculated. All measurements were blinded to groups. Experimenters were consistently performed by the same person to avoid variability.

For muscle contractility testing, mice were placed in a supine position on the platform to first assess the right hindlimb using an in vivo muscle contractility system (Aurora Scientific Inc., Canada Model 1300A Muscle). The right paw was taped to a rotating force foot plate using Transpore medical tape (3M, Maplewood, MN) connected to a dual control motor to assess plantar flexion torque. The hindlimb was secured into testing frame connected to the platform base using blunt clamps at the knee. The hindlimb and foot were positioned with the tibia and foot at a 90° angle. A pair of disposable monopolar electrodes (Natus Neurology Inc., Middleton, WI, USA) are placed subcutaneously parallel to each other. Peak twitch torque was measured during single stimulations, with the stimulus intensity adjusted to achieve a maximal twitch response. Maximum tetanic torque production was then evaluated by delivering trains of supramaximal stimuli (150 Hz for 1 s per train). A 30-s rest period was given between each stimulus. The same procedure was repeated to achieve twitch and tetanic measurements on the left leg.

The CMAP of the right gastrocnemius muscle was recorded using a Sierra Summit EMG System (Cadwell, Kennewick, WA) following sciatic nerve stimulation. In brief, mice were anesthetized with isoflurane (3 to 5% for induction, 2 to 3% for maintenance). The right hindlimb was shaved to ensure proper electrode placement and contact. Electrode gel was used on the skin underlying the ring electrodes to decrease electrical resistance (Spectra 360, Parker Laboratories, Fairfield, NJ). An active ring electrode was positioned over the gastrocnemius muscle, and a reference ring electrode was placed over the metatarsals of the right hind paw (NEUROSPEC, Stans, Switzerland). A ground electrode was placed on the animal’s tail. Two 28-gauge 1-inch monopolar needle electrodes (Ambu, Ballerup, Denmark) were inserted subcutaneously on each side of the sciatic nerve in the proximal thigh region. Sciatic nerve stimulation was delivered using a portable electrodiagnostic system (Cadwell Sierra Summit, Kennewick, WA) with a 0.1-ms pulse and 1- to 10-mA intensity. CMAP baseline-to-peak amplitudes were recorded following supramaximal stimulation. The average SMUP amplitude was calculated by averaging the peak-to-peak amplitude differences from 10 incremental submaximal responses. MUNE was then determined by dividing the maximum CMAP (peak-to-peak) response by the average SMUP.

Unilateral hindlimb grip strength was measured using the BIO-GS4 device (BioSeb, Pinellas Park, FL, USA). For grip strength evaluation, mice were positioned parallel to the tabletop, with one hind paw at a time grasping the bar. The mice were then gently pulled toward the evaluator. The average of three trials was used for each hindlimb, with measurements taken separately for both the left and right hind limbs

Recovery percentage is calculated using the following formula (*73*)

$$\%\;\text{of recovery}=\frac{\text{Injured}\;\left(\text{Treated}\right)}{\text{Healthy control}}\times 100$$

### Immunohistochemistry

After surgical procedures, sciatic nerve tissue were collected and placed in 10% neutral-buffered formalin solution (MilliporeSigma) for 72 hours at room temperature. After fixation nerves were transferred to a 30% sucrose in 1× phosphate-buffered saline (PBS) solution for an additional week at room temperature. After dehydration, the samples were frozen in optimal cutting temperature compound (OCT) (Thermo Fisher Scientific). Nerve tissue was sectioned either longitudinally or cross-sectionally at 10-μm thickness using a CryoStar NX50 cryostat (Thermo Fisher Scientific) and placed onto microscope slides (Thermo Fisher Scientific). Tissue samples were washed and permeabilized twice with a solution of 0.1% PBS Tween 20 buffer (Thermo Fisher Scientific), blocked for 1 hour at room temperature using a 5% normal goat serum (Vector Labs), and incubated with primary antibodies (table S4) overnight at 4°C. Subsequently, appropriate fluorescent-tagged secondary antibodies (table S4), and DAPI (1:10,000; Thermo Fisher Scientific) were used. Samples were mounted using VECTASHIELD Vibrance Antifade mounting medium (Vector Laboratories) and imaged using an inverted fluorescence microscope (Nikon Ti-2e) or Nikon Eclipse Ji confocal microscope.

### Imaging analysis

For the crush injury model, the amount of blood capillaries per mm^2^ and the percentage of NF positive signal of each nerve fascicle were analyzed using ImageJ-Fiji software. For linage tracing, transfection efficiency in vitro, and isograft experiments, analysis was performed using a custom-made script in the Nikon software NIS elements 6.02.01. Groups were blinded during imaging and analysis, and negative controls (no primary antibody) were used. For quantification, Z-stacks were combined using maximum intensity projection, and background signal was removed using manual thresholding based on a negative control. The percentage of myelinated axons were measured using the denoise AI tool followed by the extended depth of focus function to create a two-dimensional image. The NF and MBP channels were processed separately, thresholded, and analyzed using “having” and “not having” functions to determine the percentage of myelinated and unmyelinated axons. The having function combine the overlap of the processed NF and MBP images to calculate the percentage myelinated axons, while the not having function was used to calculate the percentage of unmyelinated axons. For the NF images, an ROI was drawn around each nerve fascicle and the total axon count per ROI was used to calculate axons/mm^2^. Last, the total number of CD31-positive cells was identified by thresholding the CD31 channel and confirming overlap with DAPI, and capillaries were counted to calculate capillaries/mm^2^.

### Statistical analysis

All statistical analyses were run using Sigma Plot version 15.0 and GraphPad Prism p Version 9.5.1. All data were graphed as means ± SEM. Data were tested for normality using Shapiro-Wilk test, and outliers were tested using ROUT method with *Q* = 1%. Normally distributed data were analyzed using one-way analysis of variance (ANOVA) with post hoc least significant difference test (Fisher least significant difference method), two-way ANOVA with Holm-Sidak, or two-tailed *t* test as appropriate. For in vitro experiments, *n* = 3 to 5 biological replicates were used and *n* = 4 to 6 for in vivo experiments. Statistical significance level was defined as *P* < 0.05 for hypothesis testing.
