# Supplementary material for: Vasculogenic tissue nanotransfection accelerates functional recovery after peripheral nerve injury
Source: Sci Adv. 2026 Apr 8;12(15):eaeb7631. doi: 10.1126/sciadv.aeb7631 (PMC13060591; doi:10.1126/sciadv.aeb7631)
Supplement: Supplementary file 1 — Figs. S1 to S11 Tables S1 to S4 [file sciadv.aeb7631_sm.pdf]

Science Advances
AAAS

Supplementary Materials for

Vasculogenic tissue nanotransfection accelerates functional recovery after peripheral nerve injury

Ana I. Salazar-Puerta *et al.*

Corresponding author: Amy M. Moore, amy.m.moore@osumc.edu; Daniel Gallego Perez, gallegoperez.1@osu.edu

Sci. Adv. **12**, eaeb7631 (2026)
DOI: 10.1126/sciadv.aeb7631

This PDF file includes:

Figs. S1 to S11
Tables S1 to S4

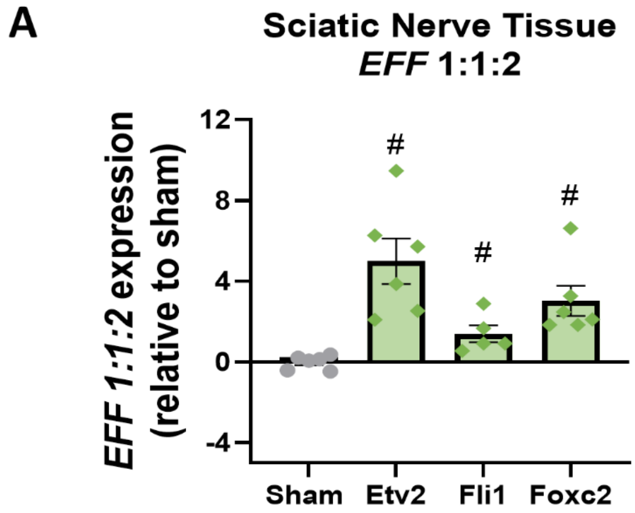

Figure S1. TNT mediates reprogramming factor delivery. (A) Gene expression analysis of sciatic nerve tissue reveals overexpression of *EFF* (1:1:2) compared to sham-treated tissue 24 hours after TNT intervention, demonstrating the feasibility of TNT-mediated delivery of *EFF* to nerve tissue using distinct factor ratios. (n=3). All error bars are shown as SEM. #Significant difference respect to the Sham with a p-value<0.05, One-way ANOVA.

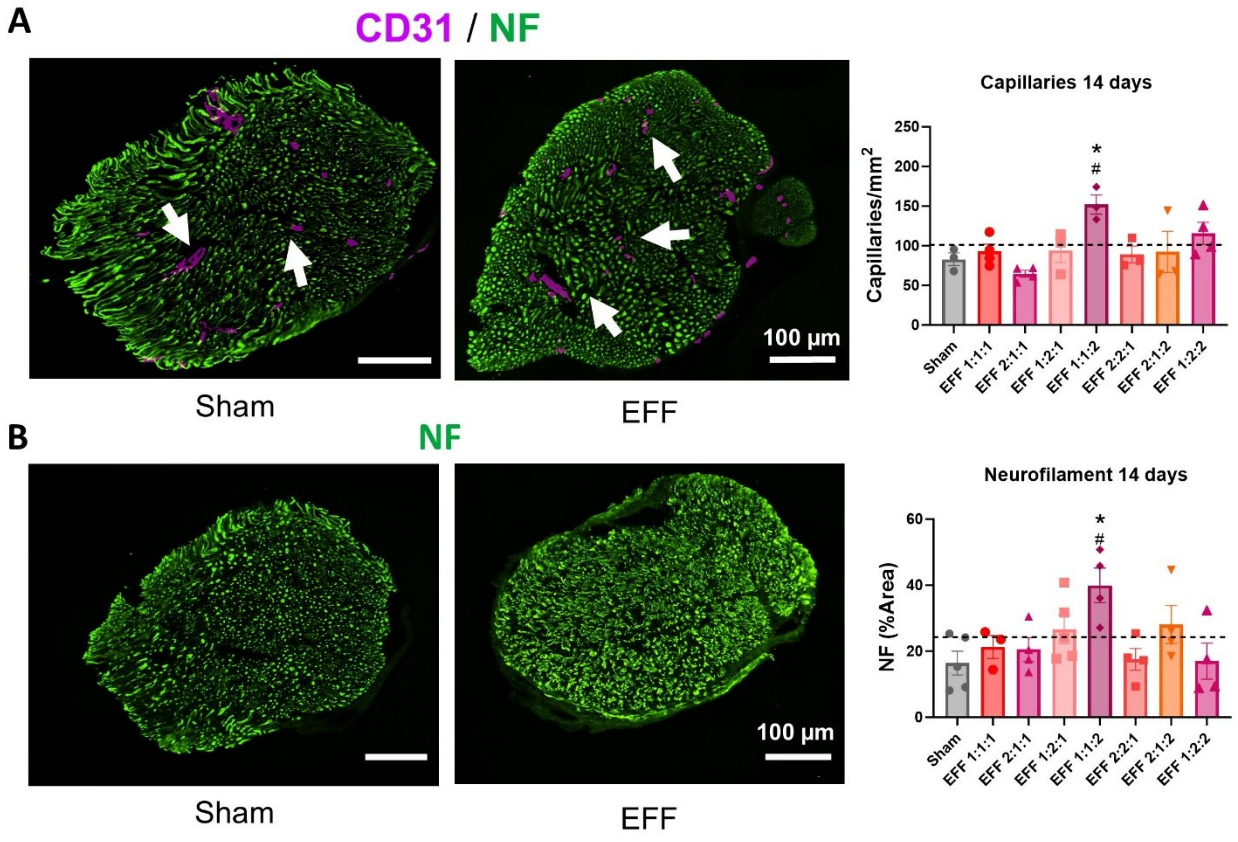

Figure S2. TNT with a vasculogenic cocktail improves vascular tissue formation in a crush injury model after 14 days. Histological analysis on cross sections evaluated 14 days post crush injury and TNT shows **(A)** a significant increase in capillary density by the expression of CD31 in the *EFF* 1:1:2 formulation compared to other EFF formulations, Sham-treated, and healthy tissue (dashed line) groups. **(B)** Moreover, analysis showed a significant difference in the expression of the axonal marker neurofilament (NF) in the *EFF* 1:1:2 group compared to all other EFF formulations, Sham-treated, and healthy tissue (dashed line). (n=3-5) All error bars are shown as SEM. #Significant difference respect to the Sham with a p-value<0.05, *Significant difference respect to *EFF* 1:1:1 with a p-value<0.05, One-way ANOVA.

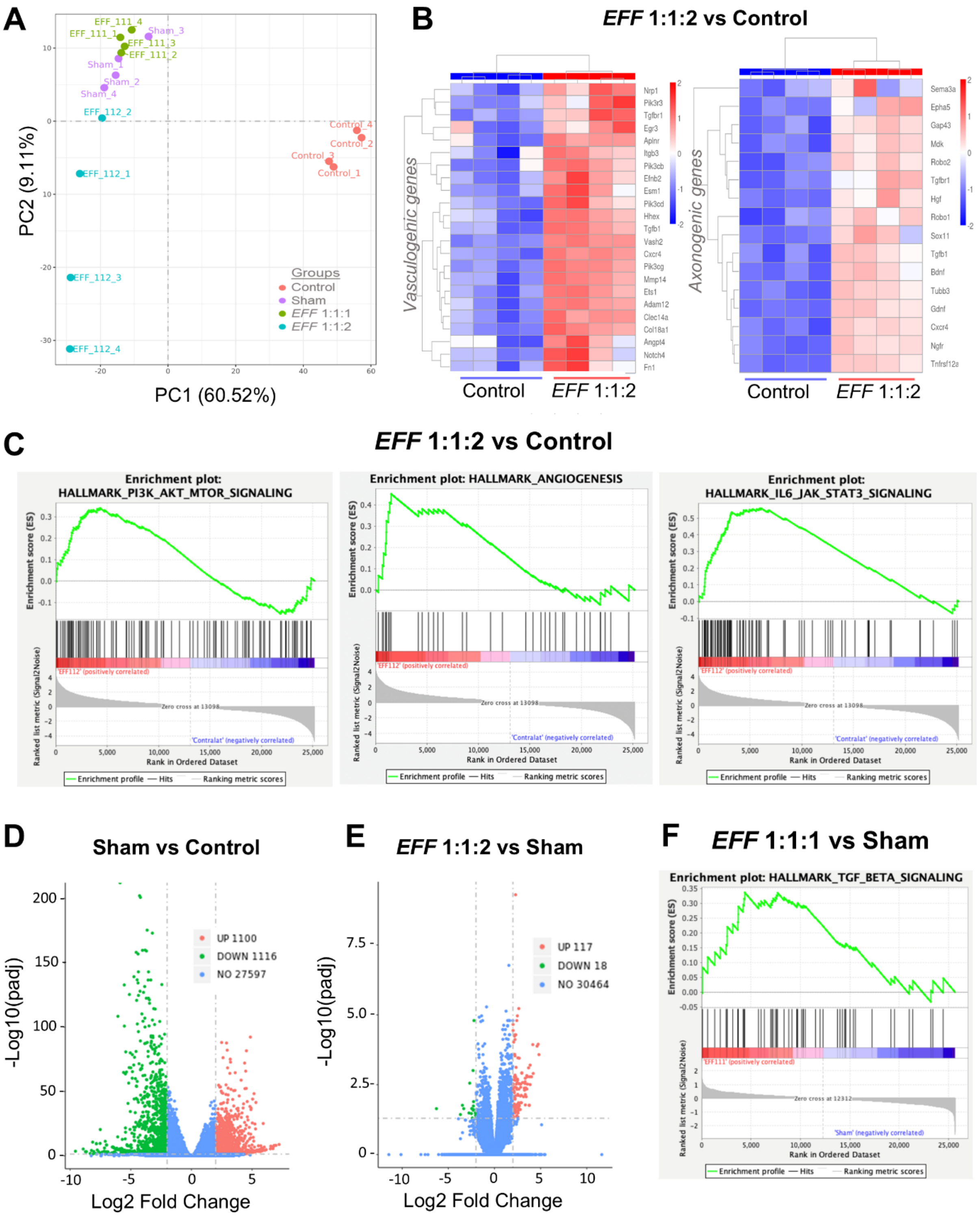

A
PC1 (60.52%)
PC2 (9.11%)
Groups
Control
Sham
EFF 1:1:1
EFF 1:1:2
B
EFF 1:1:2 vs Control
Vasculogenic genes
Axonogenic genes
Control
EFF 1:1:2
C
EFF 1:1:2 vs Control
Enrichment plot: HALLMARK_PI3K_AKT_MTOR_SIGNALING
Enrichment plot: HALLMARK_ANGIOGENESIS
Enrichment plot: HALLMARK_IL6_JAK_STAT3_SIGNALING
D
Sham vs Control
E
EFF 1:1:2 vs Sham
F
EFF 1:1:1 vs Sham
Enrichment plot: HALLMARK_TGF_BETA_SIGNALING
UP 1100
DOWN 1116
NO 27597
UP 117
DOWN 18
NO 30464
-Log10(padj)
Log2 Fold Change

Figure S3. RNA-seq analysis reveals the impact of vasculogenic reprogramming mediated by TNT in a model of crush injury. RNA-seq analysis was performed on sciatic nerve tissue 7 days after crush injury and TNT intervention, comparing the *EFF* 1:1:1 and *EFF* 1:1:2 formulations to Sham-transfected cells, and healthy tissue (control). **(A)** PCA plot shows the clustering of the samples, with control and *EFF 1:1:2* groups separated. **(B)** The most differentially expressed vasculogenic and axonogenic genes between *EFF* 1:1:2 treated tissue compared to healthy tissue are shown in the clustered heatmap. **(C)** GSEA enrichment plot highlights PIK3 AKT MTOR signaling, angiogenesis, and IL-6 Jak Stat3 signaling pathways enriched in the *EFF* 1:1:2 compared to the healthy tissue. Volcano plots illustrate the number of upregulated and downregulated genes in the **(D)** sham-treated tissue compared to the control and **(E)** *EFF* 1:1:2 TNT-treaded tissue compared to the sham-TNT group. **(F)** GSEA enrichment plot showing TGF-β signaling enriched in the *EFF* 1:1:1 compared sham-TNT. Genes with Log2FC ≥|2| and adjusted p-value ≤ 0.05 were considered significantly differentially expressed.

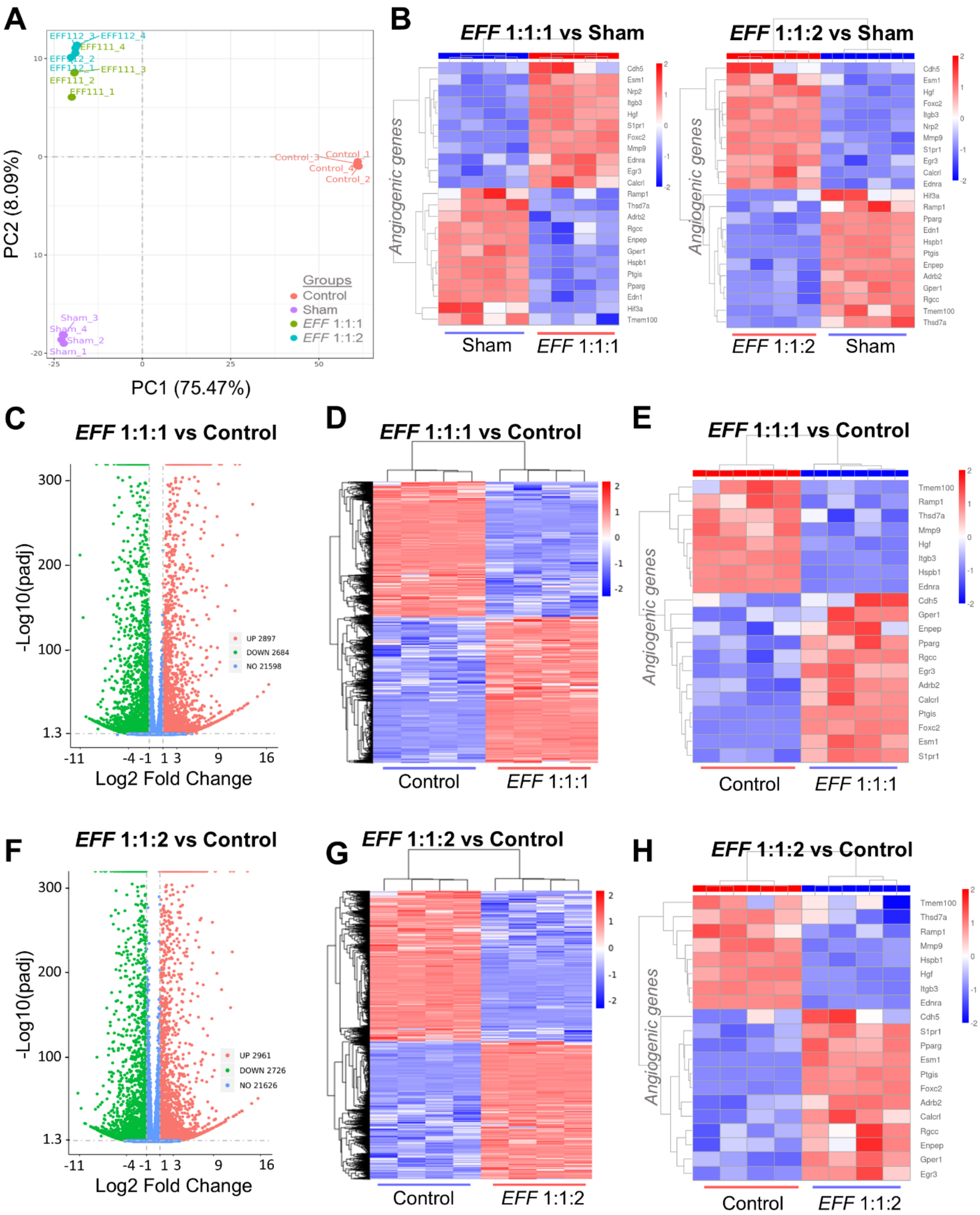
A
PC2 (8.09%)
PC1 (75.47%)
Groups
Control
Sham
EFF 1:1:1
EFF 1:1:2
B
EFF 1:1:1 vs Sham
EFF 1:1:2 vs Sham
Angiogenic genes
Sham
EFF 1:1:1
EFF 1:1:2
Sham
C
EFF 1:1:1 vs Control
-Log10(padj)
Log2 Fold Change
UP 2897
DOWN 2684
NO 21598
D
EFF 1:1:1 vs Control
Control
EFF 1:1:1
E
EFF 1:1:1 vs Control
Angiogenic genes
Control
EFF 1:1:1
F
EFF 1:1:2 vs Control
-Log10(padj)
Log2 Fold Change
UP 2961
DOWN 2726
NO 21626
G
EFF 1:1:2 vs Control
Control
EFF 1:1:2
H
EFF 1:1:2 vs Control
Angiogenic genes
Control
EFF 1:1:2

Figure S4. RNA-seq analysis shows the impact of *in vitro* vasculogenic reprogramming after electrotransfection of fibroblasts. RNA-seq analysis was performed on from fibroblasts 24 hours after transfection, comparing the *EFF* 1:1:1 and *EFF* 1:1:2 formulations to Sham-transfected and control (non-transfected) cells. **(A)** Principal component analysis (PCA) plot shows distinct clustering of samples, with control, sham, and *EFF* groups clearly separated. The strong clustering within each group indicates high similarity among biological replicates and robust transcriptomic differences between conditions. **(B)** Heatmap analysis of the most differently expressed vasculogenic genes reveals distinct clustering of samples when comparing the *EFF* 1:1:1 and *EFF* 1:1:2 with the Sham-transfected cells. Volcano plots illustrate the DEGs with significantly upregulated genes shown in red, downregulated genes in green, and non-differentially expressed genes in blue. **(C)** Transcriptomic profiles of the *EFF* 1:1:1 group compared to control cells. **(D)** Heatmap analysis of all the DEGs reveals distinct clustering of samples when comparing the *EFF* 1:1:1 with the control cells, and cluster heatmaps display the highest differentially expressed vasculogenic genes in **(E)** *EFF* 1:1:1 compared to the control. Similarly, **(F)** volcano plot showing the DEGs for the *EFF* 1:1:2 compared to control cells, **(G)** the heatmap analysis of all DEGs in the *EFF* 1:1:2 group compared to the control cells, and the **(H)** vasculogenic genes represented in the cluster heatmaps for the *EFF* 1:1:2 group compared to control cells. Genes with Log2FC $\geq|2|$ and adjusted p-value ≤ 0.05 were considered significantly differentially expressed.

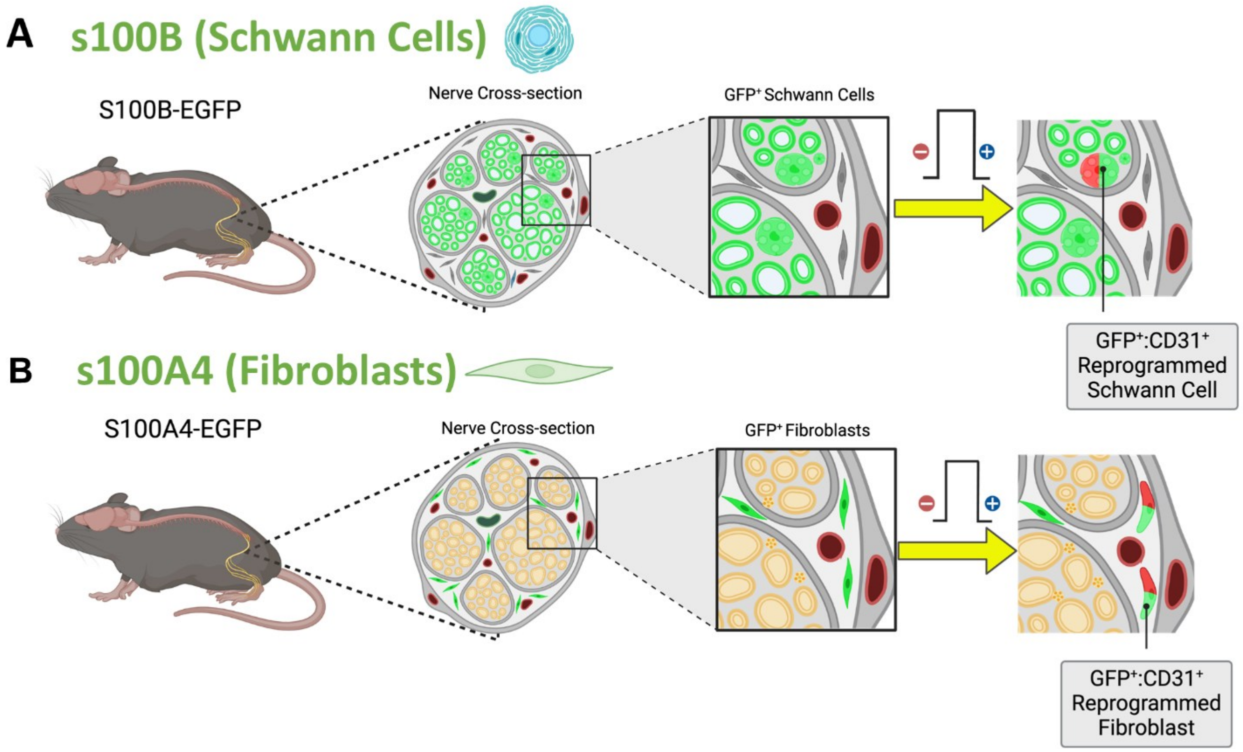

Figure S5. Transgenic mouse models to identify cell populations that are most susceptible to reprogramming. (A) Cell lineage tracing within the S100B-GFP mice sciatic nerve. In this model, nerve Schwann cells express GFP (GFP+). Similar analysis of expression of CD31 allows the identification of Schwann cells that are being reprogrammed into endothelial cells(GFP+:CD31+). **(B)** Cell lineage tracing within the S100A4-GFP mice sciatic nerve. In this model, fibroblast in a nerve cross-section express GFP (GFP+). Subsequent expression analysis of the endothelial marker CD31 (CD31+) 7 days after TNT allows the identification of fibroblasts that are being reprogrammed (GFP+:CD31+). Created in BioRender. Alzate Correa, D. (2026) https://BioRender.com/l6dfiz7.

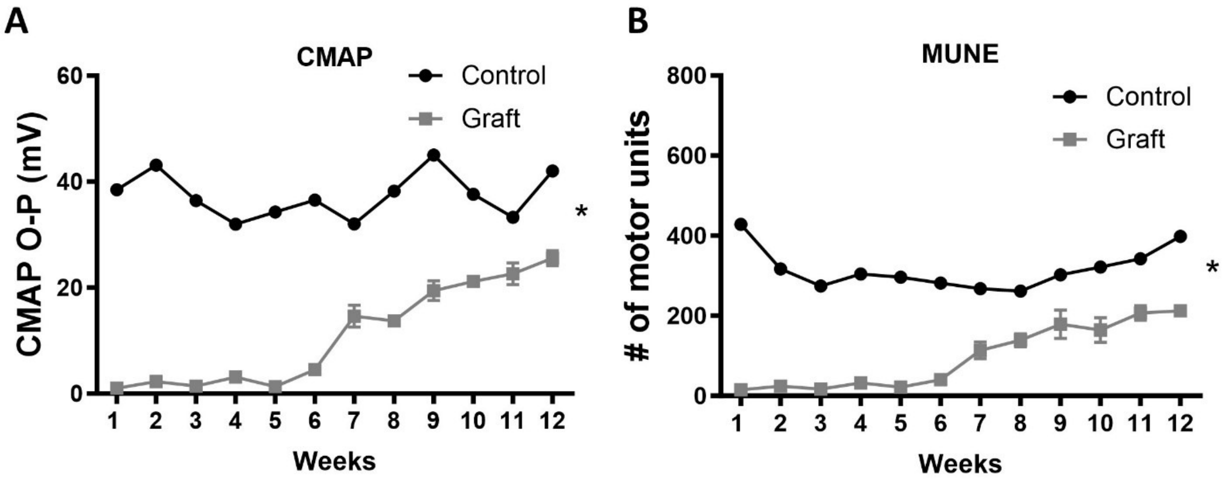

Figure S6. An optimal timeline to evaluate functional outcomes after peripheral nerve injuries and grafting procedures. Functional recovery was assessed weekly over a 12-week period using compound muscle action potential (CMAP) measurements and motor unit number estimate (MUNE) in animals subjected to grafting procedures without any TNT intervention. Early post-grafting measurements showed a substantial decrease in **(A)** CMAP amplitudes and **(B)** MUNE in mice with isografts compared to control animals. CMAP and MUNE began to recover by week 6-7 and 7-8, respectively post-grafting and showed signs of normalization by week 12. (n=4-5). * p-value<0.05, One-way ANOVA.

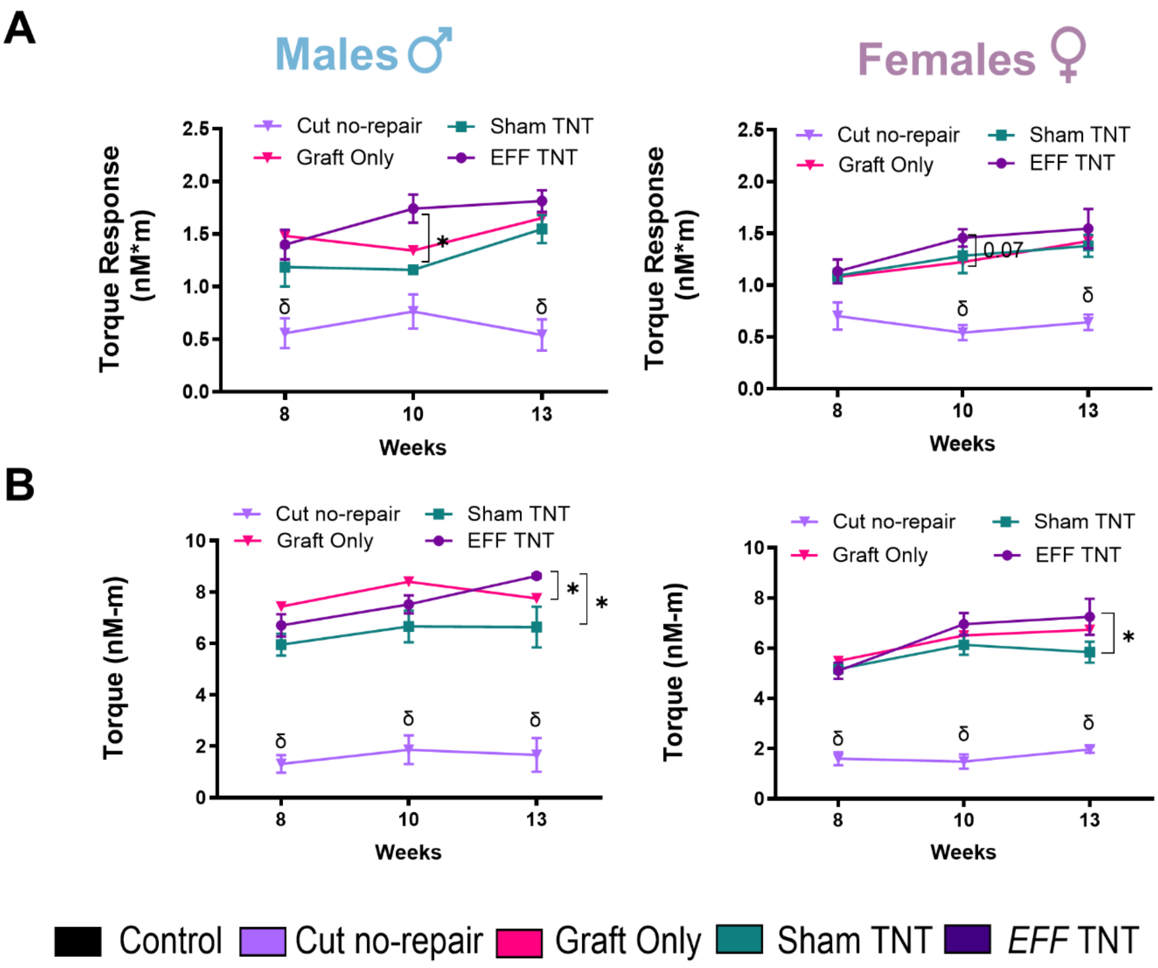

Figure S7. TNT-mediated *EFF* delivery improves functional recovery in sciatic nerve isografts with sex-associated differences: (A) *In vivo* muscle contractility assessment showed a significant increase in twitch torque in the *EFF* TNT group compared to sham TNT group at week 10 in male mice, with no differences for female mice. **(B)** Similarly, the *EFF* TNT group exhibited a significantly higher tetanic torque compared to other groups at week 13 in male mice, whereas in female mice *EFF* TNT showed a difference at week 13 compared to sham TNT. (n=6/group) All error bars are shown as SEM. # Significant difference with respect to the control, δ Significant difference with respect to cut no-repair, and * Significant difference with respect to another group. #, δ, * with a p-value<0.05. Two-way ANOVA.

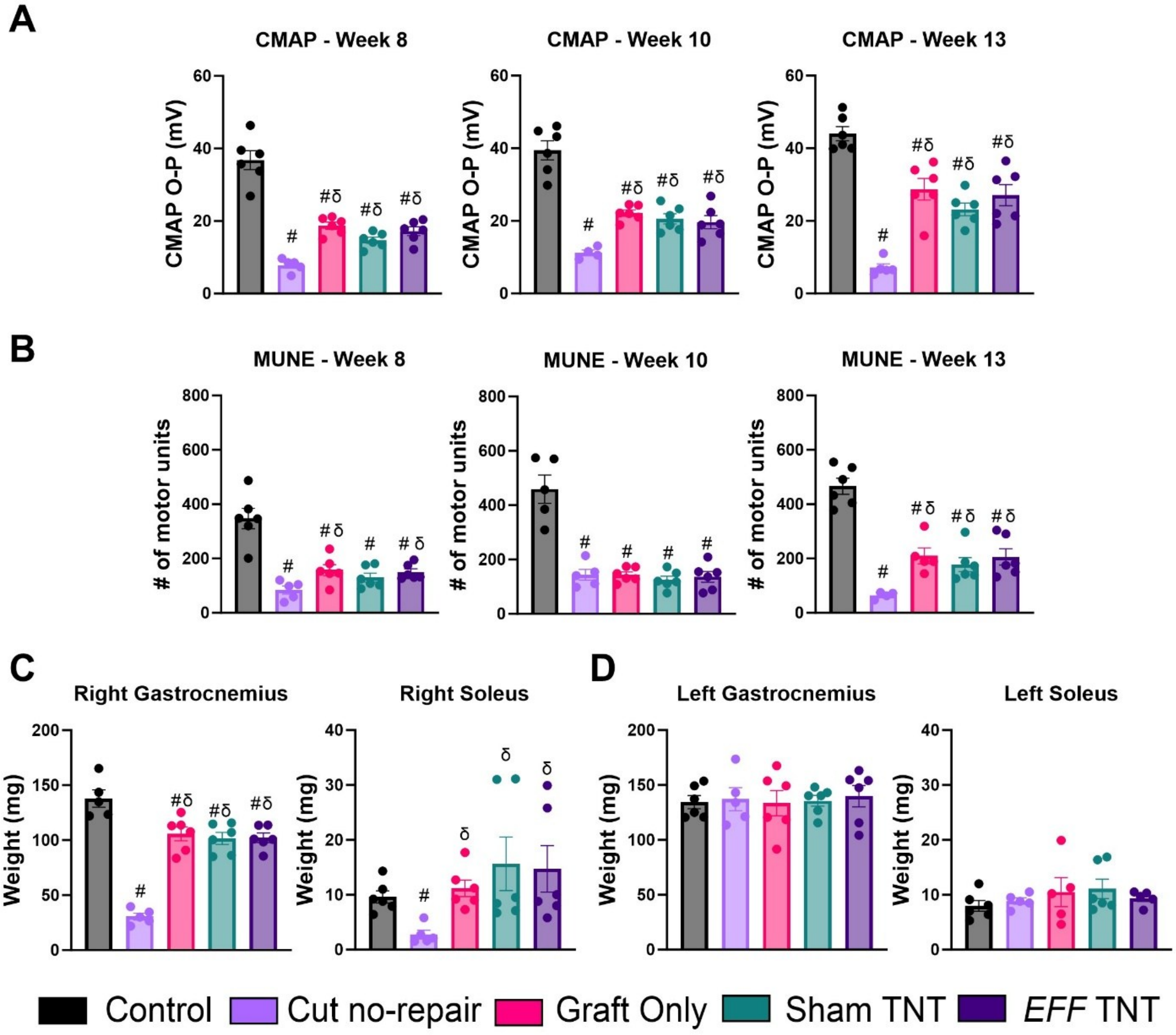
A
CMAP - Week 8
CMAP - Week 10
CMAP - Week 13
CMAP O-P (mV)
B
MUNE - Week 8
MUNE - Week 10
MUNE - Week 13
# of motor units
C
Right Gastrocnemius
Right Soleus
D
Left Gastrocnemius
Left Soleus
Weight (mg)
Control
Cut no-repair
Graft Only
Sham TNT
EFF TNT

Figure S8. Functional recovery in sciatic nerve isografts following *EFF*-TNT. Functional outcomes were evaluated over 13 weeks. The experimental groups consisted of *EFF* and sham TNT following grafting, grafting without TNT (graft only), 1 cm graft dissection without repair (cut no-repair), and healthy control animals. At early time points, nerve stimulation revealed a significant reduction in **(A)** CMAP amplitudes and **(B)** motor unit number estimate (MUNE) in all injured groups compared to controls, with gradual recovery over time in the graft only, sham TNT and *EFF* TNT groups. No significant differences were observed between the TNT-treated groups and the graft only group. At the end of the study (week 13), the weight of the gastrocnemius and soleus muscles in **(C)** the injured hindlimb (right) was significantly reduced in all injured groups compared to the control, yet partially recovered in contrast to the cut no-repair group. **(D)** No significant differences were observed among groups in the contralateral hindlimb (left). (n=6/group). All error bars are shown as SEM. # Significant difference respect to the control, δ Significant difference respect to cut no-repair, and * Significant difference respect to another group. #, δ, * with a p-value<0.05. One-way ANOVA.

A

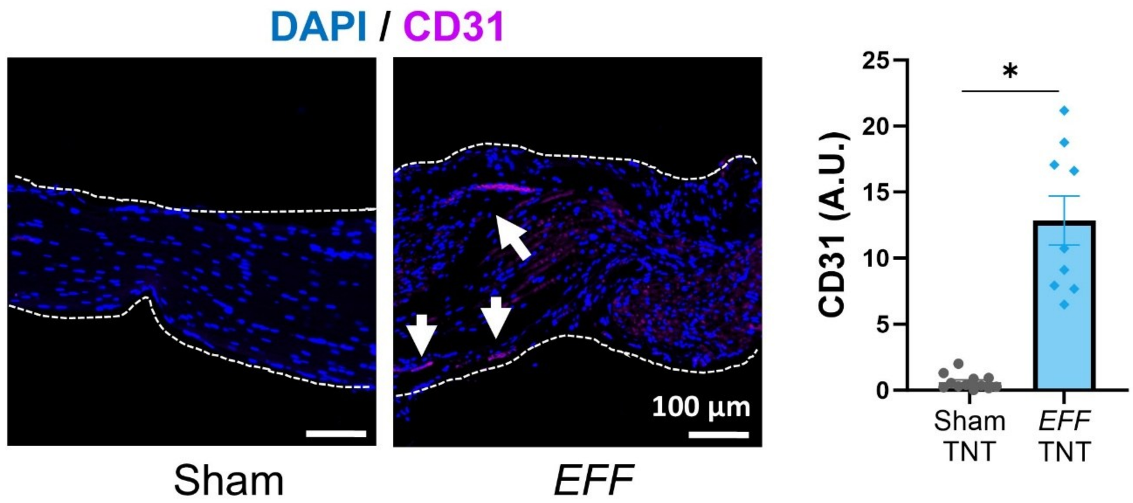

Figure S9. Vasculogenic TNT can be used in isografts as more complex models of peripheral nerve injures. (A) Segmental nerves (1 cm isografts) from donor mice were dissected, treated with TNT using *EFF* 1:1:2 or sham, and sutured into the nerve gap of genetically identical recipient mice. Representative images and histological analysis of longitudinal sections of sciatic nerve 7 days post-grafting and TNT show the overexpression of the vascular marker CD31 in the *EFF* TNT group compared to sham TNT. White arrows pointing at the blood vessel formation. (n=4) Error bars are shown as SEM. * p-value<0.05, Two-tail t-test.

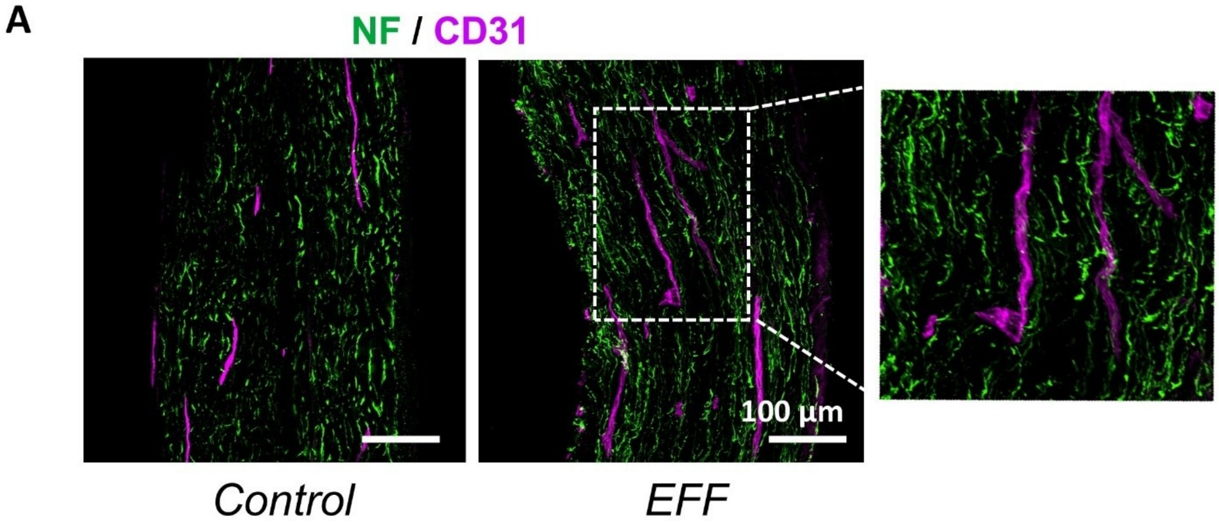

Figure S10. TNT with *EFF* improves vascular tissue formation aligned to the axons in sciatic nerve isografts after 5 weeks (A) Representative images of longitudinal sections from isografts evaluated 5 weeks post grafting and TNT interventions reveals that the newly deposited vasculature (CD31, purple) provided an aligned scaffold and promoted an organized axonal growth (NF, green) in the *EFF*-TNT group comparable to healthy sciatic nerve tissue.

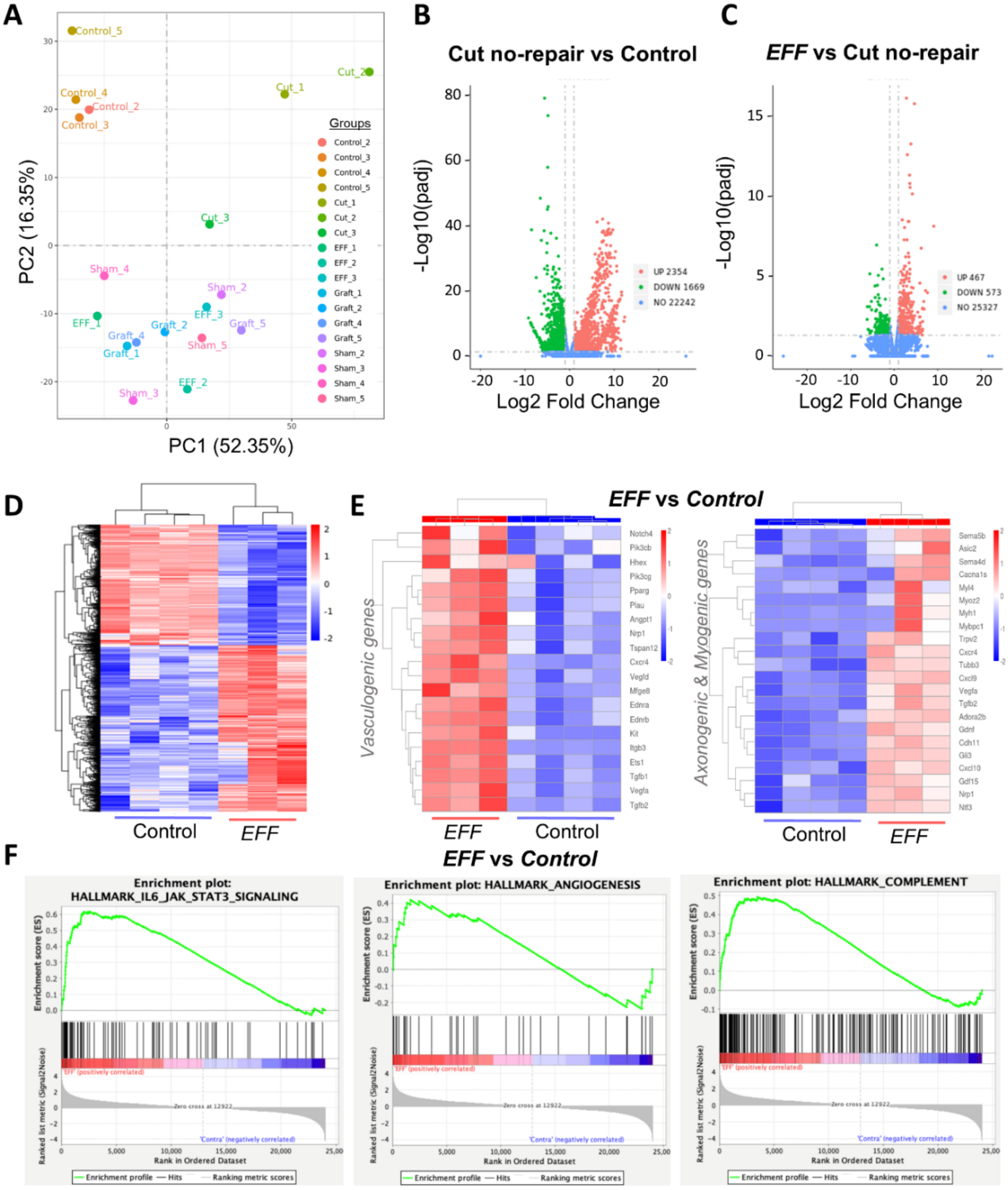
A
PC1 (52.35%)
PC2 (16.35%)
Groups
Control_2
Control_3
Control_4
Control_5
Cut_1
Cut_2
Cut_3
EFF_1
EFF_2
EFF_3
Graft_1
Graft_2
Graft_4
Graft_5
Sham_2
Sham_3
Sham_4
Sham_5
B
Cut no-repair vs Control
-Log10(padj)
Log2 Fold Change
UP 2354
DOWN 1669
NO 22242
C
EFF vs Cut no-repair
-Log10(padj)
Log2 Fold Change
UP 467
DOWN 573
NO 25327
D
Control
EFF
E
EFF vs Control
Vasculogenic genes
Notch4
Pik3cb
Hhex
Pik3cg
Pparg
Plau
Angpt1
Nrp1
Tspan12
Cxcr4
Vegfd
Mfge8
Ednra
Ednrb
Kit
Itgb3
Ets1
Tgfb1
Vegfa
Tgfb2
EFF
Control
Axonogenic & Myogenic genes
Sema5b
Asic2
Sema4d
Cacna1s
Myl4
Myoz2
Myh1
Mybpc1
Trpv2
Cxcr4
Tubb3
Cxcl9
Vegfa
Tgfb2
Adora2b
Gdnf
Cdh11
Gli3
Cxcl10
Gdf15
Nrp1
Ntf3
Control
EFF
F
EFF vs Control
Enrichment plot: HALLMARK_IL6_JAK_STAT3_SIGNALING
Enrichment plot: HALLMARK_ANGIOGENESIS
Enrichment plot: HALLMARK_COMPLEMENT
Enrichment score (ES)
Ranked list metric (Signal2Noise)
Rank in Ordered Dataset
'EFF' (positively correlated)
'Contra' (negatively correlated)
Zero cross at 12922
Enrichment profile — Hits — Ranking metric scores

Figure S11. RNA-seq analysis reveals the impact of vasculogenic reprogramming mediated by TNT in isografts. RNA-seq analysis was performed on sciatic nerve tissue 13 weeks after TNT and grafting interventions, comparing the injured groups *EFF* TNT, sham TNT, graft only, cut no-repair to the healthy control. **(A)** PCA plot shows the clustering of the samples. Volcano plots illustrating the number of upregulated and downregulated genes in the **(B)** cut no-repair compared to the control and **(C)** *EFF* TNT-treaded isografts compared to the cut no-repair group. **(D)** The unsupervised hierarchical clustering of genes expressed between the *EFF* and the control group. **(E)** The most differentially expressed vasculogenic and axonogenic genes between *EFF* treated isograft compared to healthy tissue are shown in the clustered heatmap. **(F)** GSEA enrichment plot highlights IL-6 Jak Stat3 signaling, angiogenesis, and complement activation pathways enriched in the *EFF* group compared to the healthy tissue. Genes with Log2FC $\geq|2|$ and adjusted p-value $\leq$ 0.05 were considered significantly differentially expressed.

TABLES

Table S1. Plasmid information						
**Plasmid**	**Tag**	**Type**	**Catalog Number**	**Size (kb)**	**Antibiotic resistance**	**Vector**
Etv2	Myc-DDK	Mouse Tagged ORF Clone	MR216258	5.9	Kanamycin (25 ug/mL)	pCMV6-Entry
Fli1	Myc-DDK	Mouse Tagged ORF Clone	MR225907	6.2	Kanamycin (25 ug/mL)	pCMV6-Entry
FoxC2	Myc-DDK	Mouse Tagged ORF Clone	MR221977	6.4	Kanamycin (25 ug/mL)	pCMV6-Entry
pCMV6	Myc-DDK	NA	PS100001	4.9	Kanamycin (25 ug/mL)	pCMV6-Entry
Etv2	TurboGFP	Mouse Tagged ORF Clone	MG216258	7.9	Ampicillin (100 ug/mL)	pCMV6-AC-GFP
Fli1	TurboGFP	Mouse Tagged ORF Clone	MG225907	7.9	Ampicillin (100 ug/mL)	pCMV6-AC-GFP
FoxC2	TurboGFP	Mouse Tagged ORF Clone	MG221977	8.0	Ampicillin (100 ug/mL)	pCMV6-AC-GFP
pCMV6	TurboGFP	NA	PS100010	6.6	Ampicillin (100 ug/mL)	pCMV6-AC-GFP

Table S1. Plasmid information. This table lists all plasmids used in this study, including plasmid names, Tags, type, Catalog number, size (kb), antibiotic resistance, and backbone vector.

Table S2. Different formulations of EFF			
**Formulation**	**Plasmid Concentration [µg/µl]**		
	**Etv2**	**Fli1**	**FoxC2**
EFF-111	0.05	0.05	0.05
EFF-211	0.10	0.05	0.05
EFF-121	0.05	0.10	0.05
EFF-112	0.05	0.05	0.10
EFF-221	0.10	0.10	0.05
EFF-212	0.10	0.05	0.10
EFF-122	0.05	0.10	0.10

Table S2. Different formulations of *EFF*. This table lists all *EFF* plasmid combinations tested in the study. Each formulation consists of three plasmids encoding for *Etv2*, *Fli1*, and *Foxc2*. In the formulation codes (e.g., *EFF*-111, *EFF*-112), each digit represents the concentration of the corresponding plasmid: "1" indicates 0.05 µg/µl and "2" indicates 0.1 µg/µl.

Table S3. Primer information			
**Primer**	**Hosts species**	**Assay ID**	**Vendor**
GAPDH	Mouse	Mm99999915_g1	ThermoFisher Scientific
Etv2	Mouse	Mm00468389_m1	ThermoFisher Scientific
Fli1	Mouse	Mm00484410_m1	ThermoFisher Scientific
Foxc2	Mouse	Mm00546194_s1	ThermoFisher Scientific
bFGF	Mouse	Mm00433287_m1	ThermoFisher Scientific
VEGFD	Mouse	Mm01131929_m1	ThermoFisher Scientific
CD31/ PECAM-1	Mouse	Mm01242576_m1	ThermoFisher Scientific

Table S3. Primer information. This table lists all TaqMan pre-design primers used in this study, including the primer name, host species, Assay ID, and vendor.

	Table S4. List of antibodies used for immunostaining					
**Primary Antibody**	**CAT#**	**Host species**	**Concentration**	**Vendor**	**Secondary Antibody**	**Concentration**
Neurofilament Heavy Polypeptide (NF-F)	ab4680	Chicken	1:400	abcam	Goat pAb to Chicken Alexa Fluor (488 nm)	1:200
Myelin Basic Protein (MBP)	ab218011	Rabbit	1:1000	abcam	Goat pAb to Rabbit Alexa Fluor (594 nm)	1:200
CD31/Pecam-1	ab9498	Mouse	1:500	abcam	Goat pAb to Mouse Alexa Fluor (647 nm)	1:200

Table S4. List of antibodies. This table lists all the antibodies used for immunostaining, including the primary antibody name, the catalog number (CAT #), concentration, vendor, name of the secondary antibody and its concentration.
